# Induction of Fatty Acid Oxidation Underlies DNA Damage‐Induced Cell Death and Ameliorates Obesity‐Driven Chemoresistance

**DOI:** 10.1002/advs.202304702

**Published:** 2023-12-25

**Authors:** Sunsook Hwang, Seungyeon Yang, Kyungsoo Park, Byungjoo Kim, Minjoong Kim, Seungmin Shin, Ahyoung Yoo, Jiyun Ahn, Juneil Jang, Yeong Shin Yim, Rho H. Seong, Seung Min Jeong

**Affiliations:** ^1^ Department of Biochemistry Institute for Aging and Metabolic Diseases Department of Biomedicine & Health Sciences College of Medicine The Catholic University of Korea Seoul 06591 South Korea; ^2^ Department of Systems Pharmacology and Translational Therapeutics Perelman School of Medicine University of Pennsylvania Philadelphia PA 19104 USA; ^3^ School of Biological Sciences Institute of Molecular Biology and Genetics Seoul National University Seoul 08826 South Korea; ^4^ Aging and Metabolism Research Group Korea Food Research Institute Wanju‐gun 55365 South Korea; ^5^ Division of Food Biotechnology University of Science and Technology Daejeon 34113 South Korea

**Keywords:** cell death, chemoresistance, DNA damage, fatty acid oxidation, obesity

## Abstract

The DNA damage response is essential for preserving genome integrity and eliminating damaged cells. Although cellular metabolism plays a central role in cell fate decision between proliferation, survival, or death, the metabolic response to DNA damage remains largely obscure. Here, this work shows that DNA damage induces fatty acid oxidation (FAO), which is required for DNA damage‐induced cell death. Mechanistically, FAO induction increases cellular acetyl‐CoA levels and promotes N‐alpha‐acetylation of caspase‐2, leading to cell death. Whereas chemotherapy increases FAO related genes through peroxisome proliferator‐activated receptor α (PPARα), accelerated hypoxia‐inducible factor‐1α stabilization by tumor cells in obese mice impedes the upregulation of FAO, which contributes to its chemoresistance. Finally, this work finds that improving FAO by PPARα activation ameliorates obesity‐driven chemoresistance and enhances the outcomes of chemotherapy in obese mice. These findings reveal the shift toward FAO induction is an important metabolic response to DNA damage and may provide effective therapeutic strategies for cancer patients with obesity.

## Graphical abstract

The study identifies a novel metabolic response to DNA damage involving the induction of mitochondrial fatty acid oxidation (FAO). Obesity impedes the upregulation of FAO, causing resistance to chemotherapy. Restoration of FAO overcomes obesity‐driven chemoresistance, enhancing chemotherapy outcomes. These findings underscore FAO induction as a crucial metabolic response to DNA damage, offering potential therapeutic strategies for obese cancer patients.

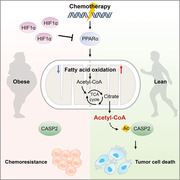

## 1 Introduction

Obesity, a physical condition defined as abnormal or excessive accumulation of fat, is increasing worldwide.^[^ ^1^ ^]^ Epidemiological studies reveal strong links between obesity and at least 13 types of cancer, including colorectal, liver, breast and prostate cancers.^[^ ^2^ ^]^ Moreover, accruing evidence suggests that obesity is closely associated with inferior clinical outcomes of cancer therapy.^[^ ^3^ ^]^ Indeed, many cancer patients with obesity suffer from chemotherapy failure, cancer recurrence, and poor prognosis.^[^ ^4^ ^]^ Thus, considering the global prevalence of overweight or obesity and the increase of cancer incidence in obese patients, it is crucial to elucidate mechanisms by which obesity influences cancer progression.

The cancer's lethal nature stems from its profound resistance to therapy. Although chemotherapy and radiation therapy, the most commonly used tools for cancer treatment, have been developed to kill cancer cells by inducing DNA damage, some cancer cells escape DNA damage‐induced cell death and develop resistance to the treatments.^[^ ^5^ ^]^ Whether innate or acquired, such therapeutic resistance is a massive obstacle to successful cancer treatment.^[^ ^6^ ^]^ Recently, systemic perturbations secondary to obesity have been implicated in enabling resistance to chemotherapy. For example, elevated circulating levels of insulin, insulin‐like growth factor (IGF‐1) and adipokines, associated with obesity‐mediated metabolic dysregulations,^[^ ^7^ ^]^ have been implicated in enabling resistance to chemotherapeutic agents.^[^ ^4a^ ^]^ It is noteworthy that obese patients recapitulate a state of persistent low‐grade inflammation characterized by increased cytokines and chemokines that induce alterations in tumor microenvironment.^[^ ^8^ ^]^ Thus, enhanced fibrosis or reactive stroma caused by increased inflammation could impair drug delivery.^[^ ^4^, ^8^ ^]^ However, it remains poorly understood whether overweight or obesity adversely affects therapeutic outcomes directly through intrinsic changes of cancer cells.

When cells are exposed to genotoxic stresses, they respond by initiating tightly coordinated signaling responses that can promote survival by inducing cell cycle arrest and DNA repair or trigger cell death to limit the accumulation of damaged cells.^[^ ^9^ ^]^ Given the indispensability of cellular metabolism for survival and growth, cells exposed to DNA damage need to make fundamental metabolic decisions of whether to upregulate biosynthesis for promoting DNA repair or not, and whether to restrict catabolic pathways for limiting proliferation or not. Surprisingly, the metabolic response to DNA damage has been largely unknown. Recent studies have shown that there is a link between metabolism and DNA damage response, by repression of glutamine metabolism to promote proper DNA repair and cell cycle arrest, and to suppress tumorigenesis,^[^ ^10^ ^]^ implying that there is a metabolic area of cellular stress response with significant therapeutic potential.

Here, we investigated how obesity modulates metabolic stress response of cancer cells to promote cell survival and chemoresistance. Using a murine model of obesity, we performed transcriptomic analyses of metabolic pathways in engrafted cancer cells after chemotherapy. We found that cancer cells in lean mice, but not in obese mice, dynamically respond to chemotherapy by upregulating fatty acid oxidation (FAO) pathway. The FAO induction promotes N‐alpha‐acetylation of caspase‐2 (CASP2), leading to apoptotic cell death. Importantly, we show that pharmacological induction of FAO alleviates obesity‐driven drug resistance and enhances therapeutic outcomes after chemotherapy. These findings may have important implications for future therapeutic approaches treating cancer patients with overweight or obesity.

## 2 Results

### 2.1 Obesity Impairs FAO Induction in Tumors after Chemotherapy

**Figure 1:**
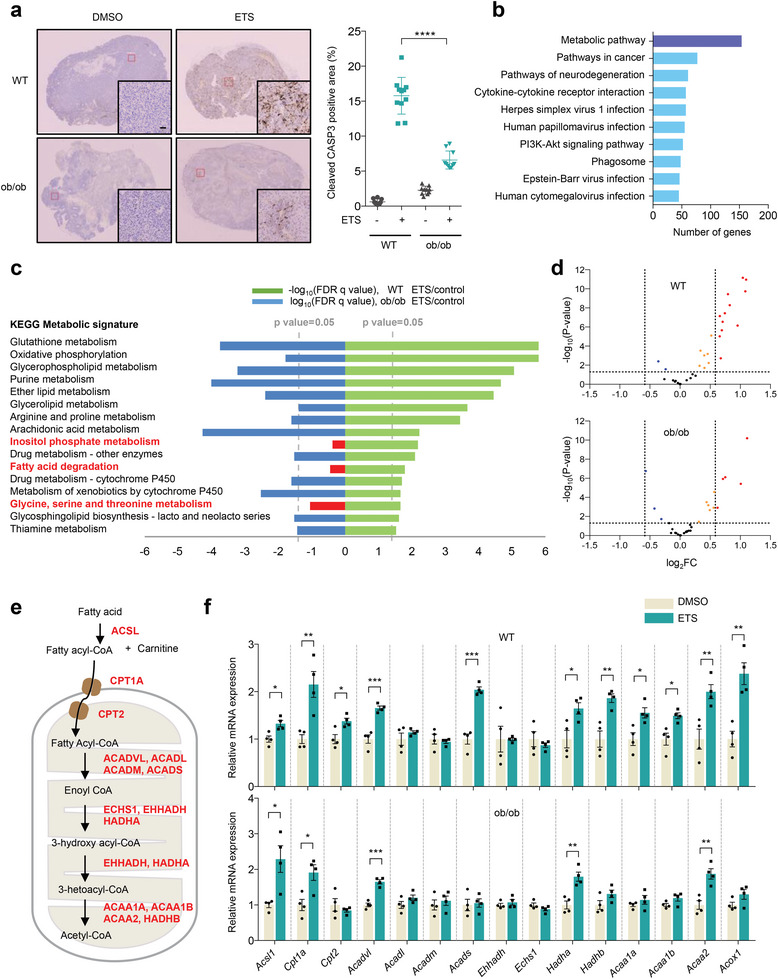
Impairment of FAO induction is related to chemoresistance in obesity. a) Immunohistochemistry of cleaved CASP3 expression pre‐ and post‐chemotherapy in B16F10 tumors from lean and obese mice. Scale bar represents 50 µm. b) Number of genes related to top 10 KEGG pathways. c) Enrichment of KEGG metabolic signature scores that are altered by chemotherapy in RNA‐seq from lean (yellow‐green bars) and obese tumors (blue bars). d) Volcano plots of differentially expressed fatty acid oxidation genes in tumors from lean (upper panel) and obese mice (lower panel) post‐ and pre‐chemotherapy (>1.5‐fold, *p* < 0.05). e) Schematic representation of mitochondrial fatty acid oxidation. f) Relative expression of FAO related genes in B16F10 tumor cells from lean (upper panel) and obese mice (lower panel); *n* = 4. Statistical analysis was based on two‐tailed Student's *t*‐test. All error bars ±SEM. *p < 0.05, **p < 0.01, ***p < 0.001, and ****p < 0.0001.

In order to investigate how obesity induces chemoresistance of tumor cells, we performed allograft tumor formation assays in wild‐type (WT) and genetic leptin‐deficient ob/ob mice using syngeneic B16F10 melanoma cells. When the tumor volume reached ≈100 mm^3^, mice were administrated with etoposide (ETS), which is one of the most common chemotherapeutic agents and has been shown to induce cell death across multiple cancers.^[^ ^11^ ^]^ As observed previously, whereas ETS treatment induced cell death in tumors from lean control mice, tumors from obese mice exhibited profound resistance to ETS (**Figure** **1**a).

To explore mechanism(s) underlying the obesity‐induced drug resistance, we performed a comparative transcriptomic analysis of tumor cells from lean and obese mice after chemotherapy. We analyzed the following four groups: lean tumor, lean tumor after ETS treatment, obese tumor, obese tumor after ETS treatment. As KEGG pathway enrichment analysis, performed on differentially expressed genes (DEGs) in lean tumors before and after chemotherapy (2418 genes; |Fc| ≥ 2 & *p*‐value cutoff < 0.05), suggested that metabolic pathways are the most significantly changed in lean tumors after chemotherapy (Figure 1b). Subsequent pathway mapping revealed that 16 KEGG metabolic signatures significantly changed in lean tumors after chemotherapy (Figure 1c). Importantly, tumors from obese mice also underwent significant changes in these pathways after chemotherapy except 3 pathways including inositol phosphate metabolism, glycine, serine and threonine metabolism, and fatty acid degradation (Figure 1c). Among these differentially regulated pathways, we reasoned that fatty acid metabolism could be most relevant to obesity given its connection to abnormalities of lipid metabolism.

To confirm that obesity abrogates the induction of fatty acid degradation after chemotherapy, we examined gene score enrichment analysis (GSEA) of DEGs in tumors from lean and obese mice pre‐ and post‐chemotherapy. The transcripts involved in fatty acid metabolism were particularly increased in lean tumors after chemotherapy, whereas these changes were not significant in obese tumors (Figure S1a, Supporting Information). Additionally, we observed that the expression of genes involved in FAO pathway was more significantly changed in lean tumors than in obese tumors after chemotherapy (Figure 1d; Figure S1b, Supporting Information). Furthermore, consistent with our transcriptomic analysis, quantitative RT‐PCR results confirmed that ETS treatment significantly increased the expression of FAO‐related genes in lean tumors, whereas the induction of these genes was markedly attenuated in obese tumors (Figure 1e,f). Taken together, our findings demonstrate that obesity impairs the induction of FAO gene signatures in tumors after chemotherapy.

### 2.2 Induction of FAO is Required for DNA Damage‐Induced Cell Death

**Figure 2:**
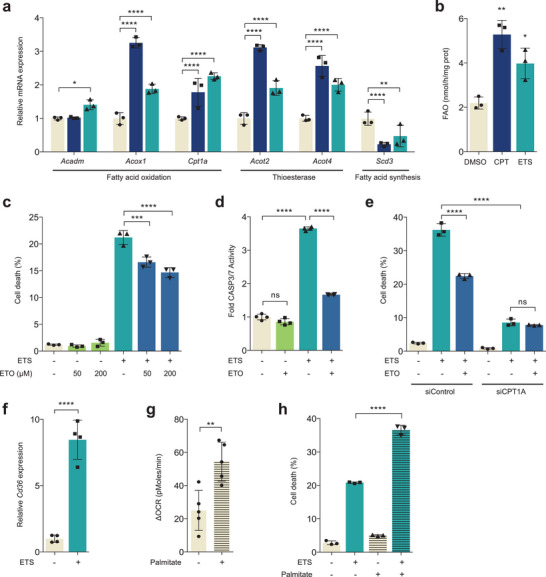
FAO induction is required for proper DNA damage‐induced cell death. a) Quantitative real time PCR of HepG2 cells showing indicated mRNA expression with CPT or ETS (*n* = 3). Statistical analysis was performed using two‐way ANOVA with Dunnett's multiple comparisons test. b) FAO was measured in HepG2 cells treated with CPT or ETS (*n* = 3). Statistical analysis was performed using one‐way ANOVA with Dunnett's multiple comparisons test. c) Cell death of B16F10 cells treated with ETS (25 µm, 40 h), ETO (50 or 200 µm, 40 h) or both (*n* = 3). Cell death was measured by propidium iodide exclusion assay. d) CASP3/7 GLO assay of B16F10 cells treated with ETS (25 µm, 24 h), ETO (50 µm, 24 h) or both (*n* = 4). e) Cell death of B16F10 cells transfected with nontargeting siRNA (siControl) or with siRNA against CPT1A (siCPT1A). Cells were treated with or without ETO (50 µm, 40 h) in the presence of ETS (25 µm, 40 h) (*n* = 3). Cell death was measured by propidium iodide exclusion assay. f) Relative *Cd36* mRNA level in B16F10 cells treated with ETS (25 µm, 24 h); *n* = 4. Statistical analysis was based on two‐tailed Student's *t*‐test. g) FAO‐dependent ΔOCR was measured in B16F10 cells pre‐treated with or without palmitate (100 µm, 6 h); *n* = 5. Statistical analysis was based on two‐tailed Student's *t*‐test. h) Cell death of B16F10 cells treated with ETS (25 µm, 40 h) in the presence of palmitate (100 µm, 40 h); *n* = 3. Statistical analysis was performed using one‐way ANOVA with Tukey's multiple comparisons test (c–e and h). All error bars ±SD. *p < 0.05, **p < 0.01, ***p < 0.001, and ****p < 0.0001.

Chemotherapies target rapidly proliferating cancer cells by directly or indirectly inducing DNA damage.^[^ ^12^ ^]^ Because the expression of FAO related genes was highly induced after ETS treatment in lean tumors but not in obese tumors, we hypothesized that FAO could serve as an important regulator of DNA damage‐induced cell death after chemotherapy. First, the relevance of FAO induction was further tested using another chemotherapeutic agent, camptothecin (CPT). In line with our in vivo results, genes involved in fatty acid degradation were significantly increased upon ETS or CPT treatment. On the contrary, genes involved in fatty acid synthesis were decreased under these conditions, suggesting that the induction of FAO related genes may be a specific metabolic response to chemotherapy and not reflective of a nonspecific metabolic crisis (**Figure** **2**a). When we directly measured FAO rates, the oxidation of palmitate, a saturated long‐chain fatty acid, was significantly increased after ETS or CPT treatment (Figure 2b).

Next to assess whether FAO affects cell death after DNA damage, we inhibited FAO using etomoxir (ETO), which can inhibit mitochondrial import of fatty acids by inhibiting carnitine palmitoyltransferase 1 family proteins (CPT1A, B and C). Notably, inhibition of FAO reduced apoptotic cell death after ETS treatment (Figure 2c,d). To further confirm the role of FAO in cell death, we suppressed CPT1A expression by using short interfering RNA (siRNA) and examined the effect on cell survival followed by ETS treatment. Knockdown of CPT1A significantly inhibited DNA damage‐induced cell death, and ETO treatment did not have addictive effects on cell death (Figure 2e; Figure S2a,b, Supporting Information). This effect was not specific to B16F10 melanoma cells. We observed similar results in immortalized embryonic fibroblasts (MEFs) (Figure S2c–f, Supporting Information). Additionally, comparable results were obtained in cells treated with other DNA damaging agents, including cisplatin or irradiation (Figure S2g,h, Supporting Information). These results demonstrate that blocking FAO protects cells from DNA damage‐induced cell death.

Next, we tested whether increasing FAO potentiates cell death in response to DNA damage. First, we found that the expression of CD36, fatty acid translocase/receptor, was markedly induced following DNA damage (Figure 2f), in line with the induction of FAO. After palmitate treatment, cells exhibited an increased oxygen consumption rate (OCR), indicating that cells were oxidizing more fatty acids (Figure 2g; Figure S2i, Supporting Information). Interestingly, cells treated with palmitate exhibited significantly elevated levels of cell death following ETS treatment (Figure 2h). We obtained comparable results in immortalized MEFs treated with palmitate or oleic acid (Figure S2j,k, Supporting Information). Finally, to test whether the relationship between FAO and cell death is unique to DNA damage or a generalizable feature of other cell death triggers, we treated cells with tumor necrosis factor α (TNFα) and cycloheximide, which induces apoptotic cell death through the engagement of death receptors. Unlike DNA damage‐induce cell death, cell death induced by activation of death receptors was not reduced by FAO inhibition (Figure S2l, Supporting Information). Taken together, these data illustrate that genotoxic stress induces FAO, which is required for DNA damage‐induced cell death.

### 2.3 DNA Damage Induces FAO through PPARα

**Figure 3:**
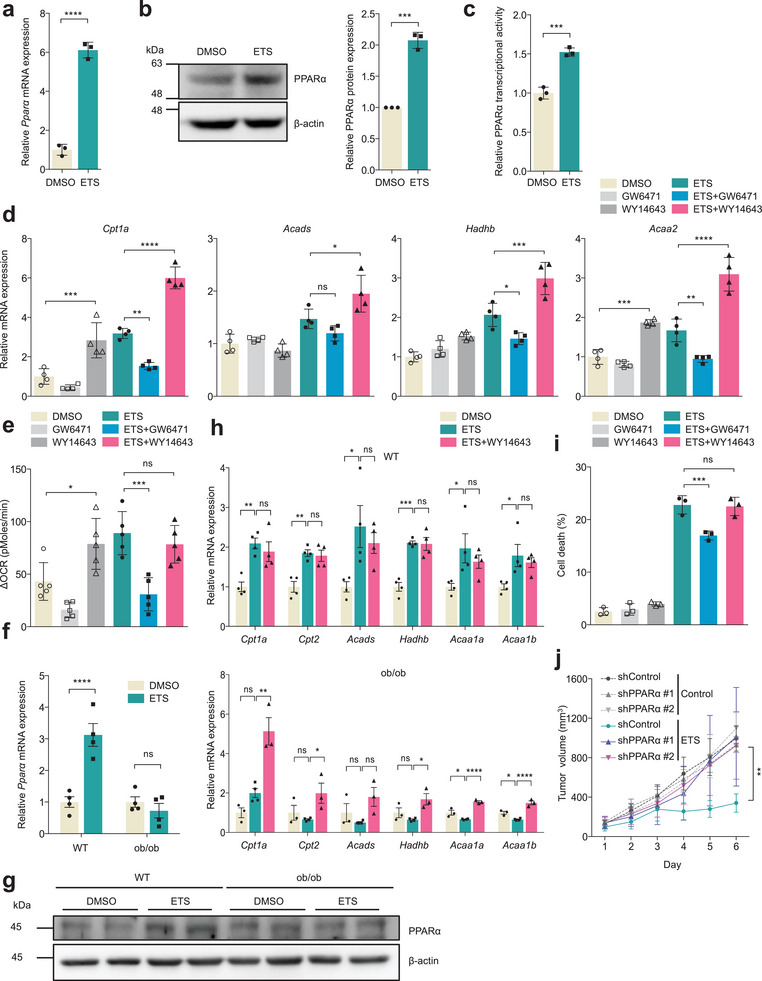
PPARα is an upstream target for DNA damage‐induced FAO induction. a–c) Relative *Ppar*α mRNA (a), protein (b), and transcriptional activity (c) levels in B16F10 cells treated with ETS (25 µm, 24 h); *n* = 3. Statistical analysis was performed using two‐tailed Student's *t*‐test. d) Relative expression of FAO related genes in B16F10 cells treated with GW6471 (25 µm, 24 h) or WY14643 (300 µm, 24 h) in the presence of ETS (25 µm, 24 h); *n* = 4. Statistical analysis was performed using one‐way ANOVA with Tukey's multiple comparisons test. e) FAO‐dependent ΔOCR of B16F10 cells treated with GW6471 (25 µm) or WY14643 (300 µm) and/or ETS (25 µm); *n* = 5. The cell medium was replaced with assay media and the cells were incubated in a CO_2_ free incubator at 37 °C for 1 h before the assay. FAO was measured using Seahorse XF Palmitate Oxidation Stress Test Kit. ETO (40 µm) was treated 15 min prior to running assay, and then Palmitate‐BSA was added to the wells immediately before running assay. Statistical analysis was performed using one‐way ANOVA with Tukey's multiple comparisons test. f,g) Relative PPARα mRNA (f) and protein (g) levels in allograft B16F10 tumors from lean and obese tumors pre‐ and post‐chemotherapy (*n* = 4). Statistical analysis was performed using two‐way ANOVA with Sidak's multiple comparisons test. h) Relative mRNA levels of FAO related genes in B16F10 tumors from lean (up) and obese (down) mice treated with or without WY14643 (40 mg kg^−1^ five times via intraperitoneal (i.p.) injection) in the presence of ETS (20 mg kg^−1^ five times via i.p. injection). *n* = 4 (DMSO, ETS, ETS+WY14643) in WT mice; *n* = 3 (DMSO, ETS+WY14643) or *n* = 4 (ETS) in ob/ob mice. Statistical analysis was performed using one‐way ANOVA with Dunnett's multiple comparisons test. i) Cell death of B16F10 cells treated with GW6471 (25 µm) or WY14643 (300 µm) and/or ETS (25 µm); *n* = 3. Statistical analysis was performed using one‐way ANOVA with Tukey's multiple comparisons test. j) Tumor volume of B16F10 cells expressing a control shRNA or two independent shRNAs to PPARα in lean mice treated with or without ETS (20 mg kg^−1^ five times via i.p. injection). Statistical analysis was performed using one‐way ANOVA with Tukey's multiple comparisons test. Error bars indicate ±SD (a‐e, and i) or ±SEM (f and h). *p < 0.05, **p < 0.01, ***p < 0.001, and ****p < 0.0001.

As our results show that DNA damaging drugs induce FAO, we sought to identify the molecular mechanism. Peroxisome proliferator‐activated receptor α (PPARα) is a critical regulator of lipid metabolism and promotes transcription of genes crucial for FAO,^[^ ^13^ ^]^ thus suggesting that DNA damage might potentially induce this metabolic response via PPARα. To test this concept, we first assessed the expression of PPARα in response to DNA damage. We observed that ETS treatment resulted in a robust increase in PPARα at mRNA level (**Figure** **3**a), as well as the protein level (Figure 3b) in B16F10 cells and multiple cell lines (Figure S3a–d, Supporting Information). Consistent with these results, the transcriptional activity of PPARα was significantly increased following DNA damage (Figure 3c).

Next, to evaluate the role of PPARα on FAO induction after DNA damage, we treated cells with either GW6471, an antagonist of PPARα, or WY14643, a synthetic agonist of PPARα. Importantly, we observed that PPARα inhibition by GW6471 abrogated the induction of FAO gene signatures upon DNA damage (Figure 3d; Figure S3e, Supporting Information). We obtained similar results in cells in which PPARα expression was reduced by siRNAs against PPARα (Figure S3f,g, Supporting Information). As the PPAR family comprises three subtypes, such as PPARα, PPARβ, and PPARγ,^[^ ^14^ ^]^ we investigated the involvement of PPARβ or PPARγ in the induction of FAO‐related genes upon DNA damage. While PPARγ knockdown had no effect on the expression of these genes, PPARβ knockdown partially reduced the induction of some genes (Figure S3f,h, Supporting Information). However, we observed that PPARβ expression remained unaffected after ETS treatment (Figure S3i, Supporting Information), implying that PPARα may play a major role in the induction of FAO upon DNA damage. Consistent with this observation, inhibition of PPARα suppressed the induction of FAO upon DNA damage (Figure 3e). Moreover, to further confirm the in vivo relevance of PPARα, we assessed whether PPARα is induced in tumors after exposure to chemotherapy. Importantly, PPARα was significantly induced in lean tumors after ETS treatment, whereas not in obese tumors (Figure 3f, g; Figure S3j, Supporting Information). These findings prompted us to further investigate whether enhancing PPARα activity rescues the defective FAO induction in obese tumors. We found that administration of WY14643 was able to rescue the impaired induction of FAO related genes in obese tumors after chemotherapy, whereas we did not detect additional effect in lean tumors (Figure 3h).

Given the function of PPARα in FAO regulation, we speculated that the PPARα‐mediated transcriptional activation of FAO‐related genes promotes cell death after chemotherapy. To test this hypothesis, we examined the effect of PPARα inhibition on cell survival after DNA damage. Notably, GW6471 treatment markedly reduced DNA damage‐induced apoptotic cell death, whereas WY14643 had minimal effect on cell death (Fig 3i; Figure S4a–d, Supporting Information). Additionally, these effects were diminished in CPT1A knockdown cells (Figure S4e, Supporting Information). Similarly, knockdown of PPARα resulted in decreased cell death following ETS treatment compared to control cells (Figure S4f,g, Supporting Information). To further confirm the importance of this pathway in DNA damage‐induced cell death, we performed allograft assays with PPARα knockdown melanoma cells to assess changes in drug‐sensitivity. Consistent with our in vitro results, PPARα knockdown led tumors to be more resistant to ETS treatment (Figure 3j). Together, these data support the idea that DNA damage induces FAO via PPARα, which promotes cell death.

### 2.4 Obesity‐Induced Hypoxia Suppresses FAO Induction after Chemotherapy

**Figure 4:**
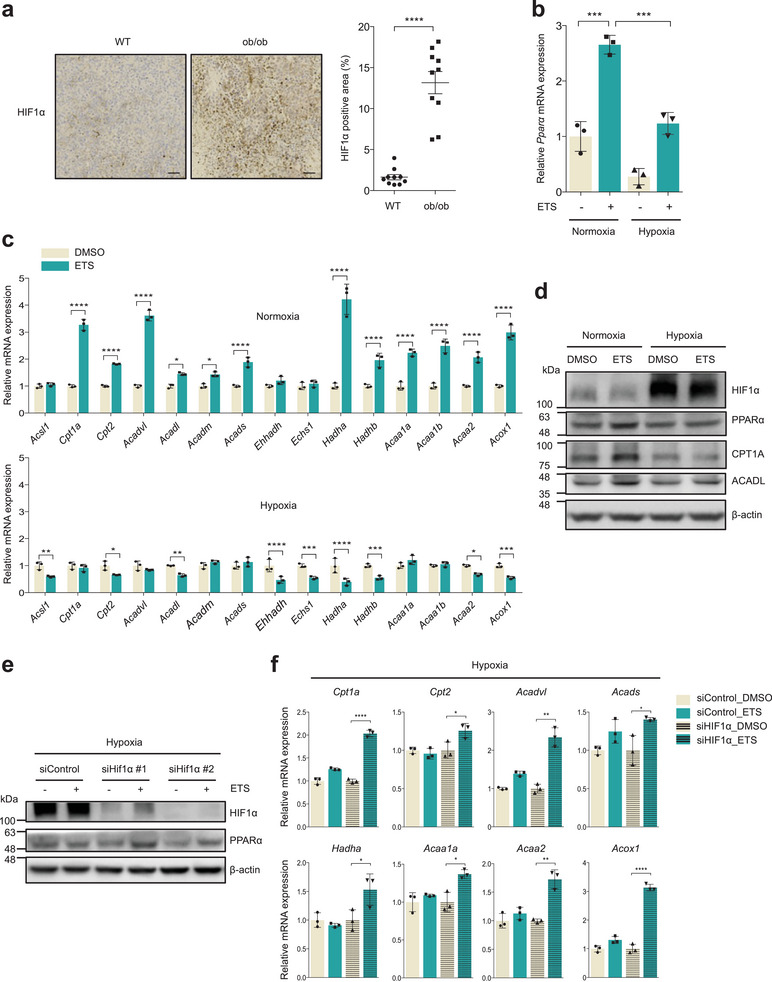
Obesity‐mediated hypoxia inhibits the FAO induction after chemotherapy. a) Immunohistochemistry of HIF1α expression in lean and obese B16F10 tumors. Scale bars represent 50 µm. Statistical analysis was performed using two‐tailed Student's *t*‐test. Error bars represent ±SEM b) Relative *Pparα* mRNA levels in B16F10 cells treated with ETS (25 µm) under normoxic or hypoxic conditions for 24 h (*n* = 3). Statistical analysis was performed using one‐way ANOVA with Tukey's multiple comparisons test. c,d) Relative mRNA (c) and protein (d) levels of FAO related genes in B16F10 cells treated with ETS (25 µm) under normoxic or hypoxic conditions for 24 h (*n* = 3). Statistical analysis was performed using two‐way ANOVA with Sidak's multiple comparisons test. e) HIF1α and PPARα expression of B16F10 cells transfected with nontargeting siRNA (siControl) or with siRNA against HIF1α (siHIF1α) under hypoxic condition. β‐actin serves as a loading control. f) Relative expression of FAO related genes in B16F10 cells transfected with nontargeting siRNA (siControl) or with siRNA against HIF1α (siHIF1α) under hypoxic condition (*n* = 3). Statistical analysis was performed using one‐way ANOVA with Tukey's multiple comparisons test. Error bars ±SD (b–f). *p < 0.05, **p < 0.01, ***p < 0.001, and ****p < 0.0001.

Because obese tumors are more hypoxic than lean tumors,^[^ ^15^ ^]^ and hypoxia‐inducible factor‐1α (HIF1α) suppresses FAO by inhibiting PPARα,^[^ ^16^ ^]^ we hypothesized that hypoxic condition occurring in obese tumors would contribute to the defect in FAO induction after chemotherapy. To test this idea, we first investigated whether obesity augments hypoxia in tumors. As has previously shown,^[^ ^15a^ ^]^ histological analysis showed that tumors from obese mice exhibited elevated levels of HIF1α relative to tumors from lean mice (**Figure** **4**a; Figure S5a, Supporting Information). To test whether hypoxia directly represses FAO induction after DNA damage, we examined the levels of FAO related genes under hypoxic conditions. ETS treatment clearly and reproducibly increased the expression of PPARα and its target genes in normoxia (Figure 4b–d; Figure S5b, Supporting Information). However, the induction of these genes was markedly blunted by hypoxia (Figure 4b–d; Figure S5b, Supporting Information). We also obtained similar results in cells treated with dimethyloxalglycine (DMOG), which stabilizes HIF1α by inhibiting prolyl hydroxylase (Figure S5c, Supporting Information). Next, to further investigate whether HIF1α directly represses FAO induction after DNA damage, we reduced HIF1α expression by siRNA targeting HIF1α and examined the expression of FAO‐related genes under hypoxic conditions. Notably, knockdown of HIF1α significantly restored the impaired induction of PPARα and FAO‐related genes after ETS treatment (Figure 4e,f; Figure S5d, Supporting Information). In conclusion, these data suggest that obesity‐mediated hypoxia opposed the FAO induction after chemotherapy.

### 2.5 FAO Regulates DNA Damage‐Induced Cell Death by Promoting N‐Alpha‐Acetylation of Caspase‐2

**Figure 5:**
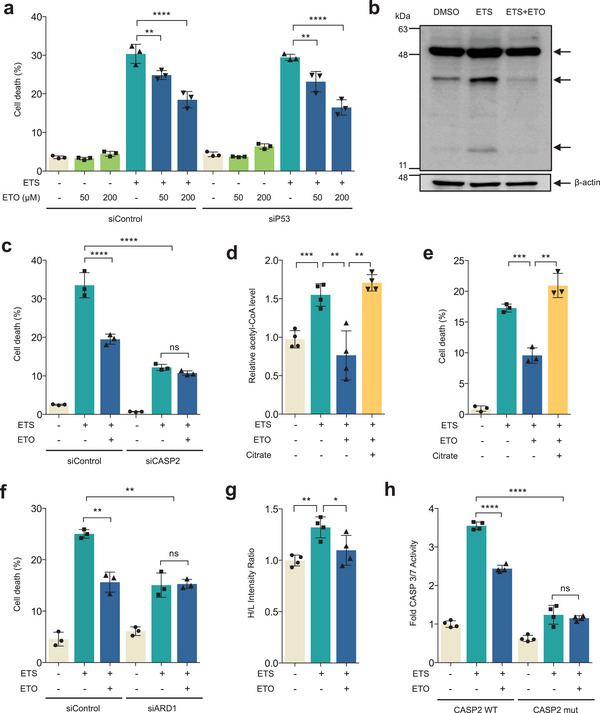
FAO adjusts DNA damage‐induced cell death by N‐alpha‐acetylation of CASP2. a) Cell death of B16F10 cells transfected with nontargeting siRNA (siControl) or with siRNA against P53 (siP53). Cells were treated with ETS (25 µm, 40 h), ETO (50 or 200 µm, 40 h) or both (*n* = 3). b) Cleaved CASP2 expression of 293T cells treated with or without ETO in the presence of ETS. β‐actin serves as a loading control. c) Cell death of B16F10 cells transfected with nontargeting siRNA (siControl) or with siRNA against CASP2 (siCASP2). Cells were treated with or without ETO (50 µm, 40 h) in the presence of ETS (25 µm, 40 h); *n* = 3. Cell death was measured by propidium iodide exclusion assay. d) Relative acetyl‐CoA levels were measured in immortalized MEFs treated with ETO (200 µm, overnight) and/or citrate (10 mm, overnight) in the presence of ETS (25 µm, overnight); *n* = 4. e) Cell death of immortalized MEFs treated with the indicated drugs (*n* = 3). Cell death was measured by propidium iodide exclusion assay. f) Cell death of immortalized MEFs transfected with nontargeting siRNA (siControl) or with siRNA against ARD1 (siARD1). Cells were treated with or without ETO (200 µm, overnight) in the presence of ETS (25 µm, overnight); *n* = 3. Cell death was measured by propidium iodide exclusion assay. g) 293T cells were transfected with FLAG‐tagged CASP2 C320G (active cysteine mutant). Transfected cells were treated with or without ETO (200 µm, 24 h) in the presence of ETS (25 µm); *n* = 4. Lysates were immunoprecipitated with FLAG magnetic beads. The bound proteins were eluted with FLAG peptide and the eluent was performed mass spectrometry. Ratios for levels of N‐terminal acetylation were determined by normalizing to total detection peptides. h) Relative CASP3/7 activity of the indicated cells. HeLa cells were infected with mock vector, hCASP2 WT, or hCASP2 A3P and selected by puromycin (2 µg mL^−1^). Infected cells were transfected with siControl or with siRNA against CASP2 (siCASP2). Both cells were treated with or without ETO in the presence of ETS (*n* = 4). All error bars ±SD. Statistical analysis was performed using one‐way ANOVA with Tukey's multiple comparisons test. *p < 0.05, **p < 0.01, ***p < 0.001, and ****p < 0.0001.

We asked how FAO regulates cell death after chemotherapy. First, to examine the involvement of p53 in FAO‐mediated cell death, we first reduced p53 expression using siRNA against p53. Notably, ETO suppressed cell death in p53 knockdown cells after ETS treatment (**Figure** **5**a; Figure S6a, Supporting Information), implying that FAO might regulate DNA damage‐induced cell death in a p53‐independent manner. Consistent with this observation, we observed similar results in p53 null PC3 human prostate cancer cells (Figure S6b, Supporting Information). Previous studies have shown that caspase‐2 (CASP2) is required for apoptosis in p53‐deficient cells.^[^ ^17^ ^]^ Thus, to investigate the contribution of CASP2 to the FAO‐mediated cell death, we examined CASP2 activity under these conditions. Interestingly, FAO inhibition markedly reduced levels of its cleaved forms after DNA damage (Figure 5b; Figure S6c,d, Supporting Information). Consistent with this observation, ETO was not able to further reduce cell death in CASP2 knockdown cells (Figure 5c; Figure S6e,f, Supporting Information), indicating that FAO might induce cell death by activating CASP2 after chemotherapy.

Previous studies have found that N‐alpha‐acetylation of CASP2 is required for its activation, which is dependent on cellular levels of acetyl‐CoA.^[^ ^18^ ^]^ Because FAO is an important source of cellular acetyl‐CoA pools,^[^ ^19^ ^]^ we hypothesized that FAO regulates DNA damage‐induced cell death by promoting CASP2 activity through acetyl‐CoA. To test this idea, we first examined whether FAO induction induces cell death via acetyl‐CoA upon DNA damage. Importantly, we found that ETS treatment elevated intracellular acetyl‐CoA levels (Figure 5d; Figure S6g, Supporting Information). The induction of acetyl‐CoA levels required FAO, as ETO treatment blocked this (Figure 5d; Figure S6g, Supporting Information). As citrate can be converted to acetyl‐CoA by ATP citrate lyase, we suspected that the decreased cell death by ETO could be restored by citrate. As expected, addition of citrate increased cellular acetyl‐CoA levels (Figure 5d; Figure S6g, Supporting Information). Notably, citrate treatment rescued the reduced DNA damage‐induced cell death by FAO inhibition (Figure 5e; Figure S6h, Supporting Information), suggesting that FAO regulates cell death by providing acetyl‐CoA.

Next, to examine the relevance of N‐alpha‐acetylation of CASP2 in the FAO‐mediated cell death, we performed a series of experiments. Arrest defective 1 (ARD1) functions as a catalytic subunit of N‐acetyltransferase protein complexes and it was reported that knockdown of ARD1 disrupts N‐alpha‐acetylation of CASP2 induced by DNA damage.^[^ ^18^ ^]^ We found that knockdown of ARD1 reduced cell death upon DNA damage, consistent with previous work (Figure 5f; Figure S6i, Supporting Information). Importantly, FAO inhibition could no longer affect cell death in ARD1 knockdown cells (Figure 5f). We next explored the direct effects of FAO inhibition on N‐alpha‐acetylation of CASP2 after DNA damage by performing mass spectrometry analysis. Indeed, we observed that the induction of N‐alpha‐acetylation of CASP2 after ETS treatment was significantly impaired by FAO inhibition (Figure 5g; Figure S6j, Supporting Information). Finally, we conducted mutagenesis of CASP2 to disrupt its N‐alpha‐acetylation by replacing the third residue of CASP2 to Pro (A3P)^[^ ^18^ ^]^ and then expressed WT or mutant CASP2 in cells after knockdown of endogenous CASP2 (Figure S6k, Supporting Information). Consistent with our model, ETO treatment suppressed DNA damage‐induced CASP3/7 activities in WT CASP2 expressed cells, whereas it had minimal effects in A3P CASP2 expressed cells (Figure 5h; Figure S6l, Supporting Information). Collectively, these data support the idea that the induction of FAO increased intracellular acetyl‐CoA levels after DNA damage, which promotes cell death by enhancing N‐alpha‐acetylation of CASP2.

### 2.6 Pharmacological Intervention for Improving FAO Rescues Obesity‐Induced Chemoresistance

**Figure 6:**
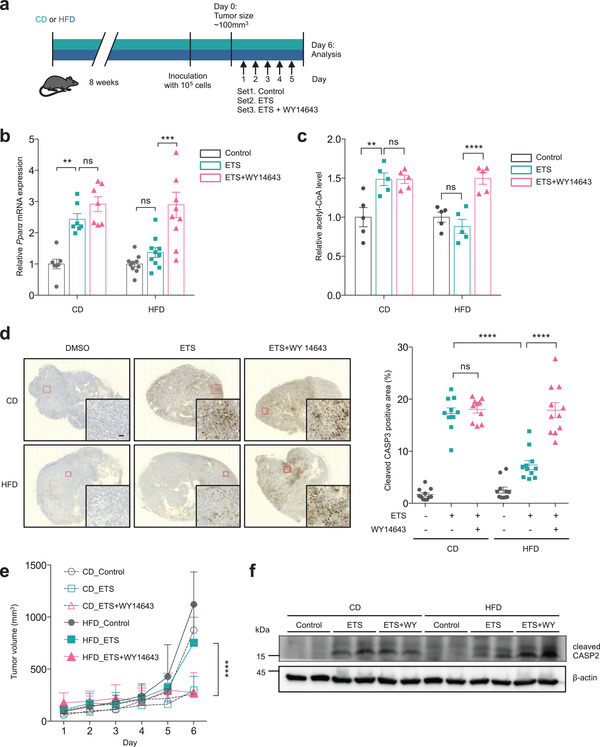
PPARα agonist improves the obesity‐induced chemoresistance. a) Schematic depicting in vivo experimental setup. b,c) b) Relative *Pparα* mRNA (*n* = 7 for CD and *n* = 10 for HFD) and c) acetyl‐CoA (*n* = 5) levels in allograft B16F10 tumors from CD‐ and HFD‐fed mice treated with or without WY14643 (40 mg kg^−1^ five times via i.p. injection) in the presence of ETS (20 mg kg^−1^ five times via i.p. injection). Statistical analysis was performed using two‐way ANOVA with Tukey's multiple comparisons test. d) Immunohistochemistry of cleaved CASP3 expression in lean and obese tumors treated with or without WY14643 in the presence of ETS. Scale bar represents 50 µm. Statistical analysis was performed using one‐way ANOVA with Tukey's multiple comparisons test. e) Tumor volume of B16F10 tumors in CD‐ and HFD‐fed mice treated with or without WY14643 in the presence of ETS. f) Immunoblot for cleaved CASP2 in lean and obese tumors treated with or without WY14643 in the presence of ETS. All error bars ±SEM. **p < 0.01, ***p < 0.001, and ****p < 0.0001.

Our results support a model wherein FAO induction is required for DNA damage‐induced cell death, which is impaired by obesity. These results led us to hypothesized that restoring FAO in tumors of obese mice might inhibit their chemoresistance. We used a diet‐induced obesity (DIO) mouse model to investigate this issue. After 8 weeks on 60% high‐fat diet (HFD), mice weighed 39.1% more than lean, chow diet (CD) control mice (Figure S7a, Supporting Information). To improve FAO induction after chemotherapy, mice subcutaneously injected B16F10 cells were treated with or without WY14643 in the presence of ETS (**Figure** **6**a). First, we tested whether our cellular findings are relevant in this model. In line with our previous results, ETS treatment induced PPARα expression in tumors from control mice, whereas HFD abrogated this induction (Figure 6b). In addition, WY14643 administration strongly induced the expression of PPARα in obese tumors after chemotherapy, whereas it had no further effect on PPARα expression in lean tumors (Figure 6b). Consistent with this, PPARα activation restored the impaired induction of FAO‐related genes in obese tumors after chemotherapy (Figure S7b, Supporting Information). We observed comparable results in ob/ob mice (Figure S7c, Supporting Information). Moreover, we demonstrated that ETS treatment increased acetyl‐CoA levels in lean tumors but not in obese tumors and PPARα activation could rescue the defect in obese tumors (Figure 6c; Figure S7d, Supporting Information). Thus, consistent with previous results, these data indicate that chemotherapy induces FAO in tumors, leading to increased acetyl‐CoA levels, and that obesity impairs this process.

To validate the effect of FAO induction on the sensitivity of tumor cells after chemotherapy, we examined tumors from CD‐ or HFD‐fed mice. As expected, chemotherapy‐induced cell death was significantly reduced in tumors from HFD‐fed mice compared with those from CD‐fed mice (Figure 6d). Importantly, the treatment of WY14643 potently synergized with ETS to induce a strong increase of cleaved CASP3 in tumors from HFD‐fed mice, resulting in reduced tumor volume in these mice (Figure 6d,e). Consistent with our previous results, WY14643 was able to increase CASP2 activity in obese tumors (Figure 6f; Figure S7e, Supporting Information). However, we found that the combination did not induce the synergistic effects in tumors from control mice (Figure 6d–f; Figure S7e, Supporting Information). Collectively, these results demonstrate that the treatment aimed at improving FAO synergizes with chemotherapy to kill tumor cells in obese mice.

## 3 Discussion

In this study, we demonstrate that FAO regulates DNA damage‐induced cell death with important implications for obesity‐induced chemoresistance. Our study shows that FAO is induced after chemotherapy, which is abrogated by obesity (Figure 1). We find that the induction of FAO is required for apoptotic cell death in response to DNA damage (Figure 2). Molecularly, DNA damage induces FAO via PPARα, which is suppressed by obesity‐mediated hypoxia (Figure 3, 4). We demonstrate that increased acetyl‐CoA production by FAO contributes to DNA damage‐induced cell death by promoting N‐alpha‐acetylation of CASP2, highlighting the potential importance of this pathway in the regulation of chemoresistance of cancer cells (Figure 5). This idea is further validated by the finding that improving FAO reduces obesity‐induced chemoresistance (Figure 6). Taken together, our studies provide important insight into the role of FAO as a regulator of DNA damage‐induced cell death and uncover a mechanism whereby obesity promotes chemoresistance of tumor cells by inhibiting this critical metabolic response to DNA damage.

Our studies reveal the significant impact of hypoxia on obesity‐driven chemoresistance. Hypoxia is a defining feature of solid tumors.^[^ ^20^ ^]^ Previously, it has been shown that hypoxia leads to resistance to cancer therapies and a higher HIF1α expression is closely associated with poor prognosis in many cancers, including ovarian, colorectal and breast cancers.^[^ ^21^ ^]^ As inhibiting HIF1α shows efficacy in restoring the decreased response to cancer therapy, it has been proposed that targeting HIF1α has therapeutic potential for efficient cancer treatment.^[^ ^22^ ^]^ In this study, we demonstrate that obesity aggravates HIF1α stabilization in tumors, inhibiting PPARα‐dependent FAO induction after chemotherapy and, thus, suppressing cell death. Importantly, we suggest that the increased FAO is not simply compensative response for maintaining energy homeostasis. Rather, FAO actively functions as an important metabolic regulator of cell death by activating the CASP2‐dependent apoptosis pathway. Thus, taken together, these data provide a molecular mechanism whereby hypoxia leads to resistance of cancers to DNA damaging therapies, such as chemotherapy and radiation.

Chemotherapy is designed to kill cancer cells.^[^ ^23^ ^]^ However, when cancer cells experience non‐lethal doses, they can remain to survive or proliferate, or drug‐resistant subpopulations can be emerged by clonal expansion.^[^ ^24^ ^]^ Thus, appropriate chemotherapy doses are crucial for treatment efficacy. Because chemotherapy entails systemic treatment with cytotoxic agents, optimal doses for normal‐weight patients are often not enough for overweight or obese patients.^[^ ^25^ ^]^ Although doses of drugs are determined by using the body surface area, many obese patients received suboptimal chemotherapy dosing, such as due to concerns regarding toxicity, which leads to limited treatment efficacy in obese patients.^[^ ^26^ ^]^ However, given that obese patients are more likely to develop several complications including peripheral neuropathy and that chemotherapy can cause neurotoxicity, selecting optimal chemotherapy doses for obese patients is challenging.^[^ ^26^, ^27^ ^]^ We propose that enhanced FAO significantly attenuates obesity‐induced drug resistance. As a proof of concept, we show that PPARα activation synergizes with chemotherapy to induce robust cell death in obese tumors without increasing chemotherapy doses. Thus, our findings may help to develop chemotherapeutic approaches for cancer patients with overweight or obesity.

Although our current work highlights the potential role of FAO induction by PPARα in ameliorating obesity‐driven chemoresistance, it does not preclude the involvement of other PPARα‐dependent pathways. As PPARα plays crucial roles in cellular lipid homeostasis,^[^ ^28^ ^]^ it may regulate DNA damage‐induced cell death via other branches of lipid metabolism or other targets. In this regard, fatty acid degradation is closely associated with lipolysis process and lipophagy formation. As these lipid‐related pathways lead to the regulation of cell death through lipotoxicity or lipid peroxidation‐induced ferroptosis,^[^ ^29^ ^]^ it will be important for future work to examine how these pathways coordinately regulates cell survival and death upon genotoxic stress. Additionally, it has been reported that DNA damage induces mitochondrial biogenesis through the activation of AMP‐activated protein kinase (AMPK).^[^ ^30^ ^]^ Thus, an increased mitochondrial mass may contribute to the enhanced FAO after DNA damage. On the other hand, we demonstrate that FAO induction in cancer cells contributes to cell death after chemotherapy. However, it is important to note that in vivo cancer tissues consist of several types of cells, including cancer cells, cancer stem cells, inflammatory cells, adipocytes, and various related tissue cells.^[^ ^31^ ^]^ This heterogeneity of the cancer microenvironment could affect the FAO‐mediated cell death. In addition, metabolic alteration is a key feature of many cancer cells, which may influence this pathway. For example, metastatic cancer cells exhibit an aberrant dependency on FAO.^[^ ^32^ ^]^ Previously, it has been shown that FAO inhibition re‐sensitize radiation‐resistant nasopharyngeal carcinoma cells to radiation‐therapy.^[^ ^33^ ^]^ As therapy‐resistant cancer cells acquire resistance, they often undergo metabolic shifts or remodeling, potentially affecting the FAO‐dependent DNA damage‐induced cell death. Last, there may be cancer‐type‐specific differences, as different cancer cells have different metabolic dependencies.

This study identifies an essential role of FAO in the DNA damage response by regulating cell death. As the DNA damage response is essential for maintaining cellular and organismal homeostasis, dysregulations of this pathway cause stress‐induced premature senescence and are frequently reported in accelerated aging syndromes.^[^ ^34^ ^]^ We found that cells with the defective FAO induction upon DNA damage resist to cell death. As senescence is an alternative decision for cells when they bypass death,^[^ ^35^ ^]^ surviving cells can enter a senescence‐like state. Intriguingly, recent studies have reported that defects or dysregulations of FAO are observed in senescent cells or in aged tissues.^[^ ^36^ ^]^ Thus, it is plausible that defects in this metabolic response involving FAO regulation could be one of the causes of the undesirable accumulation of senescent cells during aging.

In sum, our work illustrates that FAO is intimately involved in cellular response to genotoxic stress, in part by regulating DNA damage‐induced cell death. Moreover, we found that improving this metabolic response could alleviate obesity‐driven chemoresistance. Given the essentiality of metabolism in cell fate decision and cancer progression, these findings may hold profound implications for developing novel therapeutic strategies for cancer as well as for elucidating the cellular metabolic response to stress.

## 4 Experimental Section

### Cell Lines

Primary MEFs were immortalized using SV40 large T antigen at passage 3. MEFs, B16F10, 293T, HeLa, HepG2 and PC3 cells were cultured in Dulbecco's modified Eagle's medium [DMEM high Glucose (with 4500 mg L^−1^ D‐glucose and L‐glutamine and without sodium pyruvate)] (WelGENE, LM001‐07) supplemented with 10% FBS (Gibco, 16000–044) and penicillin/streptomycin (Biowest, L0022‐100). The PPARα knockdown cell lines were generated via lentivirus. shRNAs for PPARα (TRCN0000026028 and TRCN0000026046) were purchased from Sigma‐Aldrich. The sequences for shRNAs were: GCTCCTTTGATATGATACTTT for shPPARα #1 (TRCN0000026028), GCTATGAAGTTCAATGCCTTA for shPPARα #2 (TRCN0000026046). Lentiviral supernatants were produced by transfecting 293T cells using Lipofectamine 2000 (Invitrogen). B16F10 cells were infected with viral supernatant supplemented with 8 mg mL^−1^ polybrene. Puromycin (2 µg mL^−1^) was used to select shRNA or control expressing cells. Cells were cultured at 37 °C and 5% CO_2_ in a humidified incubator. Hypoxic cultures were performed in Thermo Fisher Forma Anaerobic system (Thermo fisher scientific, 1029), 5% CO_2_ and less than 1% O_2_.

### Mice

All animal experiments were performed according to the National Institutes of Health's Guide for the Care and Use of Laboratory Animals, with approval of the Animal Experiment Ethics Committee of the Catholic University of Korea, College of Medicine. 4‐week‐old C57BL/6J and ob/ob mice were purchased from Central Lab Animal Inc (Korea). 4‐week‐old C57BL/6 mice were purchased from Orient Bio (Korea). Mice were maintained in a specific pathogen‐free facility with 12 h light and 12 h dark cycles.

### Mouse Tumor Models

6‐week old C57BL/6J or ob/ob mice were injected subcutaneously in the abdominal flank with 1 × 10^5^ B16F10 cells. Tumor volumes were measured using the following ellipsoid formula: volume (mm^3^) = 0.5 × length × (width^2^), where length indicates the long diameter and width is the short diameter. When the tumor volumes reached ≈100 mm^3^, ETS (20 mg kg^−1^) and WY14643 (40 mg kg^−1^) were intraperitoneally administered five times everyday. For a diet‐induced obesity mice model, 5‐week old C57BL/6 mice were fed normal CD or 60% HFD (Research Diets, D12492) for 8 weeks. After 8 weeks of CD or HFD feeding, mice were injected subcutaneously in the abdominal flank with 1 × 10^5^ B16F10 cells. When the tumor volumes reached ≈100 mm^3^, ETS (20 mg kg^−1^) and WY14643 (40 mg kg^−1^) were intraperitoneally administered five times everyday. A day after a course of treatment, mice were sacrificed for collection of tumor tissue.

### Antibody

β‐actin (Genetex, GTX109639), CPT1A (Abcam, ab234111; Cell signaling, 12252S), ACADL (Proteintech, 17526‐1‐AP), PPARα (Abcam, ab215270; Abcam, ab24509; Milipore, MAB3890), CASP2 (Enzo, 11B4), Cleaved CASP3 (Cell signaling, 9664S), and HIF1α (Cayman, 10006421).

### Reagent

ETS (E1383), ETO (E1905), GW6471 (G5045), WY14643 (C7081), DMOG (D3695), CPT (C9911), Cisplatin (PHR1624), Cycloheximide (C4859), Palmitic acid (P0500), Oleic acid (O1008), Sodium citrate (PHR1416), Actinomycin D (A1410), Anti‐FLAG M2 Magnetic Beads (M8823), and FLAG Peptide (F3290) were purchased from Sigma‐Aldrich. Lipofectamine RNAiMAX (13778150) and Lipofectamine 2000 (11668019) were purchased from Invitrogen.

### siRNAs and Transfection

20 nm siRNAs were transfected in cells right after being seeded at a density of 30–50% confluency depending on experiments using Lipofectamine RNAiMAX (Invitrogen, 13778150) according to the manufacturer's protocols. Cells were harvested ≈24–40 h post‐transfection as described in the figure legends. Human CASP2 siRNA (hs.Ri.CASP2.13.1), mouse HIF1α siRNA #2 (mm.Ri.Hif1a.13.1), and mouse PPARα siRNA (mm.Ri.Ppara.13.1, mm.Ri.Ppara.13.3) were purchased from Integrated DNA Technologies. The oligomer sequences were: AAGAAGGCAAGCCAACCUUAGCUAC for human siCASP2 (hs.Ri.CASP2.13.1); CAGGGTCACTTGGAAGACTTA for mouse siCASP2; GGAGCGACUCUUCAAUACUUCCCGCAUCC for mouse siCPT1A; CCAACACCCUCAACUUUCAGAUCAG for mouse siARD1; GUGGAUAGCGAUAUGGUCAUU for mouse HIF1α siRNA #1; GAUAUGUUUACUAAAGGACAAGUCA for mouse HIF1α siRNA #2 (mm.Ri.Hif1a.13.1); GAAAGUCCCUUAUCUGAAGAAUUCT for mouse PPARα siRNA #1 (mm.Ri.Ppara.13.1); GACCAAGUCACCUUGCUAAAGUACG for mouse PPARα siRNA #2 (mm.Ri.Ppara.13.3); CACCUCACUGCAUGGACGAUCUGUU for mouse P53 siRNA; GGAGCAUCCUCACCGGCAA for mouse PPARβ siRNA; GCCCUUUACCACAGUUGAU for mouse PPARγ siRNA.

### RNA Extraction and Quantitative RT‐PCR

RNA was isolated from cell lines or tumor tissues using Trizol (Invitrogen, 15596026) according to the manufacturer's instructions. 0.5 µg of total RNA was reverse‐transcribed using the PrimeScript RT Master Mix (Takara, RR036A). Quantitative RT‐PCR was performed using SYBR Green I Master (Roche, 04887352001) on a Lightcycler 480 (Roche). The level of gene expression was normalized to β‐actin. The primer sequences used for qPCR are listed in Supplementary Table (Supporting Information).

### Western Blotting

Cells were lysed with EZ‐RIPA lysis buffer (ATTO, WSE‐7420) supplemented with protease inhibitor cocktail (Roche, 11873580001). Cell lysates were separated by SDS‐PAGE and transferred onto nitrocellulose membranes (GE Healthcare) or polyvinylidene fluoride membranes (GE Healthcare). The membranes were blocked for 1 h in 5% nonfat milk or 3% BSA and then incubated with primary antibodies overnight at 4 °C. After washing five times in 1X TBST, the membranes were incubated with species‐specific secondary antibodies and detected using LAS 4000.

### Flow Cytometric Measurement of Cell Death

Cells at less than 70% confluence were treated with drugs. After treatment, cells were harvested by trypsinization, pelleted by centrifugation, and resuspended in PBS. The measurement of cell death was performed by flow cytometry using propidium iodide (PI) staining and/or annexin V staining (BD Biosciences, 556547).

### Caspase 3/7 GLO

Cells were plated into 96‐well plates at 2000 cells per well in 100 µL of growth media. Casp2 WT and CASP2 mutant (A3P) stable cell lines were transfected with siCASP2 for 24 h and plated into 96‐well plates at 2000 cells per well in 100 µL of growth media. The following day, cells were treated with ETS, ETO, or both. 100 µL of Caspase‐GLO 3/7 reagent (Promega, G8091) were added to each well of white‐walled 96‐well plate and incubated at room temperature for 30 min. After incubation, the luminescent signal was detected using plate‐reading luminometer.

### Acetyl‐CoA Measurement

Cellular acetyl CoA level was detected by PicoProbe Acetyl CoA assay kit (Abcam, ab87546) according to the manufacturer's instructions. Briefly, 2 × 10^6^ cells were harvested, resuspended in 500 µL of assay buffer on ice, and then homogenized using dounce homogenizer on ice. After centrifugation at 10000 g for 10 min at 4 °C, perchloric acid was added to a final concentration of 1 m in the supernatants. After centrifugation, the supernatants were neutralized with KOH. The samples were added CoA Quencher and then added Quencher Remover. The samples were supplied with reaction mix (assay buffer, substrate mix, conversion enzyme, enzyme mix, and PicoProbe) and measured using microplate reader (Ex/Em = 535/587 nm). The acetyl‐CoA standard was used in the range of 0–100 pm.

### Cloning

Human FLAG‐CASP2 WT overexpression vector was constructed by PCR‐amplifying human CASP2 with cloning primers containing NgoMIV restriction site using PfuUltra II Fusion HS DNA polymerase (Agilent) from human cDNAs. FLAG‐hCASP2 A3P was generated by PCR amplifying using the same reverse cloning primer of FLAG‐CASP2 WT cloning and the forward primer replaced the third residue of CASP2 with proline. The digested insert was ligated into pBabe vector using ligase (Takara, 2011A). FLAG‐CASP2 C320G mutant was generated using mutagenesis kit (Agilent, 200523) according to the manufacturer's instructions. Primer sequences for cloning were: GCCGGCATGGCGGCGCCGAGCGCGGGG and GCCGGCTCACTTGTCGTCATCGTCTTTGTAGTCTGTGGGAGGGTGTCCTGG for human CASP2 WT with C‐terminal FLAG; GCCGGCATGGCGCCGCCGAGCGCGGGGTCTT and GCCGGCTCACTTGTCGTCATCGTCTTTGTAGTCTGTGGGAGGGTGTCCTGG for human CASP2 A3P with C‐terminal FLAG; GTTCTTCATCCAGGCCGGCCGTGGAGATGAGACTG and CAGTCTCATCTCCACGGCCGGCCTGGATGAAGAAC for human CASP2 C320G mutagenesis.

### Mass Spectrometry

293T cells were transiently transfected with FLAG‐tagged CASP2 overexpression vector containing active cysteine mutation (C320G) to reduce cell death. Cells were treated with or without ETO in the presence of ETS for 24 h and then lysed with 0.2% Tween 20 and 0.2% Triton X‐100 buffer with protease inhibitors and phosphatase inhibitors. FLAG‐tagged CASP2 was immunoprecipitated with FLAG M2 magnetic beads (Sigma‐Aldrich, M8823) overnight at 4 °C. The beads were washed five times with lysis buffer and additionally washed three times with PBS. The bound proteins were eluted with FLAG peptide. The Eluants were analyzed by mass spectrometry performed by the Proteomics Core Facility at the School of Biological Sciences in Seoul National University.

### Immunohistochemistry

Tumors were fixed in 4% paraformaldehyde and embedded in paraffin. Paraffin‐embedded tumors were sectioned at 3 µm thickness and section slides were deparaffinized, rehydrated, and incubated with citrate buffer (ImmunoBioScience, AR‐6544‐05) at 95 °C for antigen retrieval. The slides were incubated in 1.4% hydrogen peroxide/methanol for quenching endogenous peroxidase activity and blocked with 2.5% normal horse serum (Vector Laboratories, MP‐7401) for 1 h. The slides were incubated with anti‐cleaved Caspase 3 (Cell signaling, 9664S) or anti‐HIF1α antibodies (Cayman, 10006421) at 4 °C overnight. The following day, the slides were incubated with a micropolymer HRP‐conjugated secondary antibody (Vector Laboratories, MP‐7401) for 1 h at room temperature. Antigens were revealed by DAB (Vector Laboratories, SK‐4100) and then the slides were counterstained with EASYSTAIN Harris Hematocylin (YD diagnostics, S2‐5). Finally, the slides were dehydrated in increasing concentrations of ethanol, mounted with Permount mounting medium (Fisher Scientific, SP 15–100), and imaged using Axio Scan.Z1 (ZEISS).

### Fatty Acid Oxidation

HepG2 cells were cultured overnight in media containing 100 µm palmitate and 1 mm carnitine. Cells were pulsed with 1.7 µCi [9,10(n)−3H]palmitic acid (GE Healthcare) for 2 h. To analyze the released 3H2O, the medium was collected and eluted using ion exchange columns packed with DOWEX 1 × 2‐400 ion exchange resin (Sigma).

### Oxygen Consumption Rate Measurement

OCR was measured using the Seahorse XFe96 Extracellular Flux Analyzer (Seahorse Bioscience). Briefly, cells were seeded in XFe96 cell culture microplates and cultured in DMEM supplemented with 10% fetal bovine serum, 100 U mL^−1^ of penicillin, and 100 µg mL^−1^ of streptomycin at 37 °C in a humidified 5% CO_2_ atmosphere. Thereafter, the cells were pre‐treated with BSA, PA, GW, WY, and/or ETS for 6 h. Afterward the cell medium was replaced with assay medium and the cells were transferred to a CO_2_ free incubator and maintained at 37 °C for 1 h before the assay. FAO was measured using Seahorse XF Palmitate Oxidation Stress Test Kit (Agilent, 103693‐100) according to the manufacturer's protocols. ETO (40 µm) was added to the respective cells 15 min prior to running the assay in the Seahorse analyzer. Finally, BSA or Palmitate‐BSA was added to the appropriate wells immediately before running the assay. The injection ports were loaded with 1.5 µm oligomycin A, 2 µm carbonyl cyanide‐4‐(trifluoromethoxy) phenylhydrazone (FCCP), and 1 µm rotenone/antimycine A. Following calibration, OCR measurements were recorded at every cycle of mixing (3 min) and measurement (3 min) for 75 min. Results were analyzed using Seahorse XFe96 Wave software (Agilent). FAO‐dependent OCR (ΔOCR) was determined as the (OCR untreated with ETO – OCR treated with ETO).

### PPARα Transcription Factor Assay

PPARα’s transcriptional activity was measured using the PPARα Transcription Factor Assay Kit (ab133107, Abcam) according to the manufacturer's instructions. Nuclear lysate was added to a 96‐well plate that was coated with a specific double‐stranded DNA (dsDNA) containing the peroxisome proliferator response element (PPRE) sequence. Samples were incubated overnight at 4 °C, and after five times washing, the primary antibody targeting PPAR‐α was added for 1 h. HRP‐conjugated secondary antibodies were added following five times washes. After adding the developing buffer for 45 min, stop solution added the samples. The samples were measured the absorbance at 450 nm.

### RNA Sequencing

RNA was isolated from tumor tissues of C57BL/6J and ob/ob mice pre‐ and post‐chemotherapy using Trizol reagent according to the manufacturer's instructions. RNA sequencing libraries were prepared using TruSeq Stranded mRNA LT Sample Prep Kit and sequenced with 101 bp paired‐ends on an Illumina platform at MacrogenTM (Seoul, South Korea). Reads were aligned to the mm10 genome using HISAT v2.1.0. HISAT utilizes two types of indexes for alignment (a global, whole‐genome index and tens of thousands of small local indexes). These two types of indexes were constructed using the same BWT (Burrows–Wheeler transform)/ a graph FM index (GFM) as Bowtie2. Because of its use of these efficient data structures and algorithms, HISAT generates spliced alignments several times faster than Bowtie and BWA widely used. Aligned reads were performed transcription assembly using StringTie v2.1.3b. Expression profiles were extracted using FPKM and TPM values and differentially expressed genes (DEGs) were identified based on |fold change| > 2 and the *p*‐value < 0.05. Gene set enrichment analysis was performed based on KEGG database. RNA seq data were deposited in the Gene Expression Omnibus under accession code GSE208632.

### RNA‐Sequencing Analysis

The relative abundances of genes were measured in Read Count using StringTie. The statistical analysis was performed to find differentially expressed genes using the estimates of abundances for each gene in samples. Genes with one more than zeroed Read Count values in the samples were excluded. To facilitate log2 transformation, 1 was added to each Read Count value of filtered genes. Filtered data were log2‐transformed and subjected to TMM normalization. Statistical significance of the differential expression data was determined using exactTest using edgeR and fold change in which the null hypothesis was that no difference exists among groups. False discovery rate (FDR) was controlled by adjusting p value using Benjamini–Hochberg algorithm. For DEG set, hierarchical clustering analysis was performed using complete linkage and Euclidean distance as a measure of similarity. Gene‐enrichment and functional annotation analysis and pathway analysis for significant gene lists were performed based on gProfiler and KEGG pathway.

### Statistical Analyses

Sample numbers and sizes were indicated by dots. Statistical significance was calculated using two‐tailed Student's *t*‐test to compare two groups and one‐way ANOVA or two‐way ANOVA to compare multiple comparisons. All experiments were performed at least two independent experiments and the sample number of each experiment were more than 3. All statistical analyses and *p* values are described in the figure legends. Statistical analyses were performed using GraphPad Prism 6 software. Error bars represented as mean ±SD or ±SEM. *p < 0.05, **p < 0.01, ***p < 0.001, and ****p < 0.0001.

### Data Availability

RNA‐seq data were deposited in the Gene Expression Omnibus under accession code GSE208632. All other data supporting the findings of this study are available by request to the corresponding author.

## Conflict of Interest

The authors declare no conflict of interest.

## Author Contributions

S.M.J. designed the study and wrote the main manuscript text. S.H. performed the experiments, analyzed the data, and wrote the manuscript. S.Y., K.P., B.K., M.K., S.S., A.Y., J.A., and J. J. performed the experiments and reviewed the manuscript. Y.S.Y. and R.H.S. reviewed the manuscript.

## Supporting information

Supporting Information

## Data Availability

The data that support the findings of this study are available on request from the corresponding author. The data are not publicly available due to privacy or ethical restrictions.
