# Supplementary material for: Induction of Fatty Acid Oxidation Underlies DNA Damage‐Induced Cell Death and Ameliorates Obesity‐Driven Chemoresistance
Source: Adv Sci (Weinh). 2023 Dec 25;11(10):2304702. doi: 10.1002/advs.202304702 (PMC10933680; doi:10.1002/advs.202304702)
Supplement: Supplementary file 1 — Supporting Information [file ADVS-11-2304702-s001.pdf]

ADVANCED SCIENCE
Open Access

Supporting Information

for *Adv. Sci.*, DOI 10.1002/advs.202304702

Induction of Fatty Acid Oxidation Underlies DNA Damage-Induced Cell Death and Ameliorates Obesity-Driven Chemoresistance

*Sunsook Hwang, Seungyeon Yang, Kyungsoo Park, Byungjoo Kim, Minjoong Kim, Seungmin Shin, Ahyoung Yoo, Jiyun Ahn, Juneil Jang, Yeong Shin Yim, Rho H. Seong and Seung Min Jeong**

Supporting Information

Induction of fatty acid oxidation underlies DNA damage-induced cell death and ameliorates obesity-driven chemoresistance

*Sunsook Hwang, Seungyeon Yang, Kyungsoo Park, Byungjoo Kim, Minjoong Kim, Seungmin Shin, Ahyoung Yoo, Jiyun Ahn, Juneil Jang, Yeong Shin Yim, Rho Hyun Seong, and Seung Min Jeong**

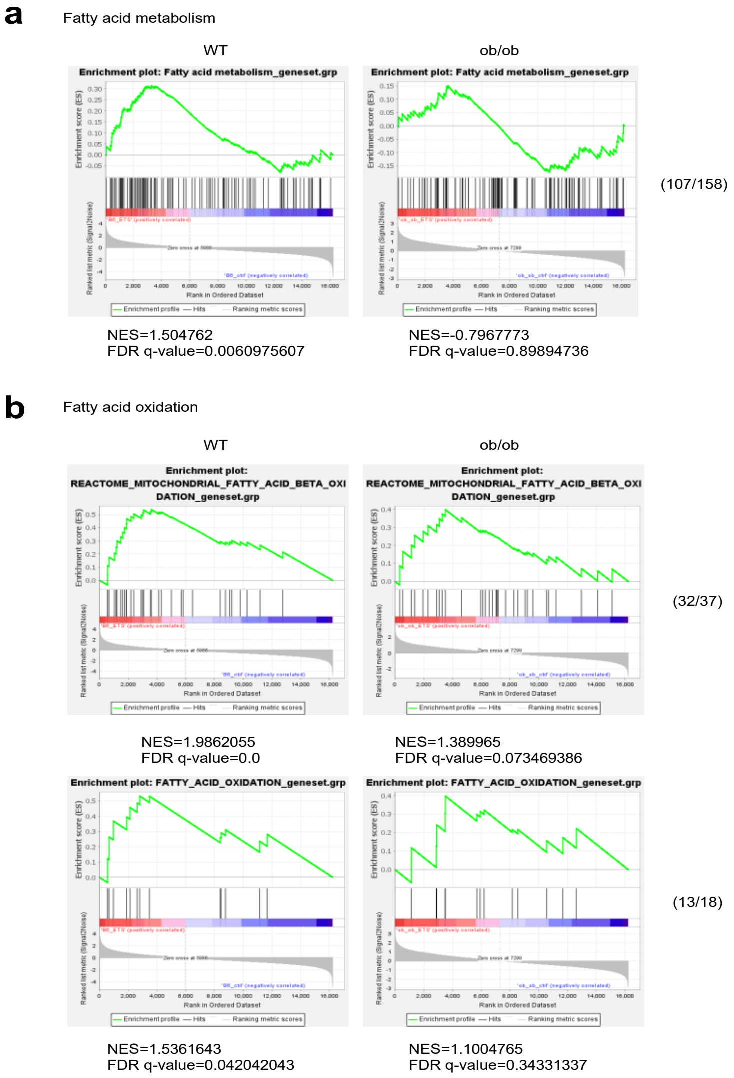

Figure S1. FAO is increased in lean tumors upon chemotherapy, but these changes are not significant in obese tumors.

(a and b) Gene set enrichment analysis (GSEA) enrichment score curves of post-chemotherapy vs pre-chemotherapy from lean tumors and obese tumors. The analysis demonstrates (a) fatty acid metabolism and (b) fatty acid oxidation of post- vs pre-chemotherapy in tumors from lean and obese mice.

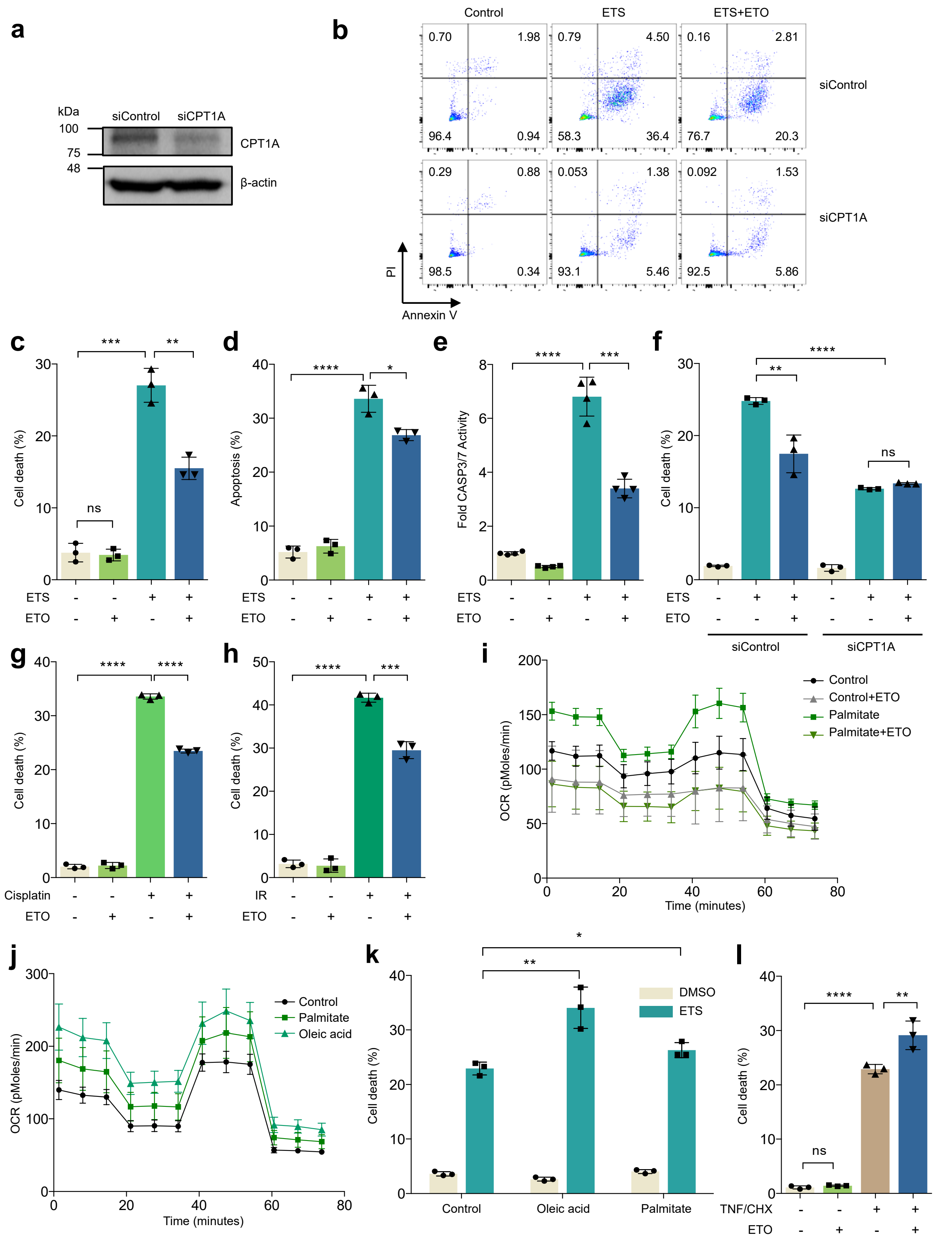

Figure S2. FAO is induced through DNA damage-induced cell death.

(a) CPT1A protein levels in cells expressing a control siRNA or siRNAs to CPT1A. (b) Representative FACS plots of B16F10 cells transfected with nontargeting siRNA (siControl) or with siRNA against CPT1A (siCPT1A). Cells were treated with or without ETO (50 µM, 40 h) in the presence of ETS (25 µM, 40 h). Cell death was measured by propidium iodide and annexin V co-staining. (c-e) Cell death of immortalized MEFs treated with ETS (25 µM, overnight), ETO (200 µM, overnight) or both (n = 3). PIpropidium iodide exclusion assay (c), Annexin V staining (d), and Casp3/7 GLO assay (e) measured cell death. (f) Cell death of immortalized MEFs transfected with nontargeting siRNA (siControl) or with siRNA against CPT1A (siCPT1A). Cells were treated with or without ETO (200 µM, overnight) in the presence of ETS (25 µM, overnight) (n = 3). Cell death was measured by propidium iodide exclusion assay. (g) Cell death of immortalized MEFs treated with cisplatin (40 µM, 24 h), etomoxir (200 µM, 24 h) or both (n = 3). Cell death was measured by propidium iodide exclusion assay. (h) Cell death of immortalized MEFs exposed to 30 Gy of IR and then treated with or without ETO (200 µM) for 3 days (n = 3). Cell death was measured by propidium iodide exclusion assay. For c-h, statistical analysis was performed using one-way ANOVA with Tukey's multiple comparisons test. (i) OCR for B16F10 cells treated with or without palmitate (100 µM, 6 h). The cell medium was replaced with assay media and the cells were incubated in a CO_2 free incubator at 37°C for 1 h before the assay. FAO was measured using Seahorse XF Palmitate Oxidation Stress Test Kit. ETO (40 µM) was treated 15 min prior to running assay, and then Palmitate-BSA was added to the wells immediately before running assay. (j) OCR for immortalized MEFs treated with of oleic acid (200 µM, 12 h) or palmitate (200 µM, 12h). (k) Cell death of immortalized MEFs treated with ETS (25 µM, overnight) in the presence of oleic acid (200 µM, overnight) or palmitate (200 µM, overnight). (l) Cell death of immortalized MEFs treated with TNF/CHX (10 ng/ml of TNFalpha and 1 mg/ml of cyclohexamide, 24 h), ETO (200 µM, 24 h) or both (n = 3). All error bars ±SD. $^{*}p < 0.05$, $^{**}p < 0.01$, $^{***}p < 0.001$ and $^{****}p < 0.0001$.

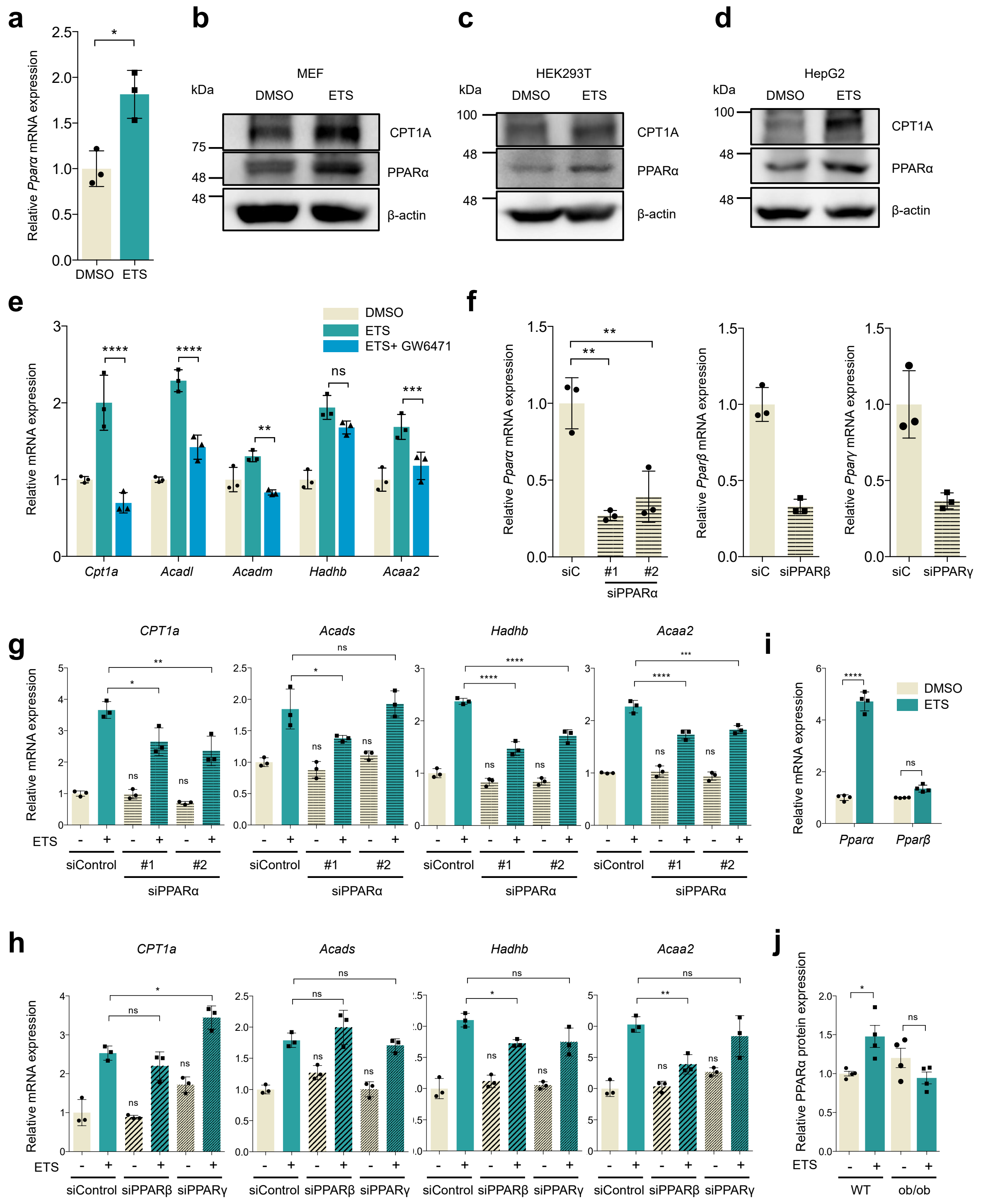

Figure S3. PPARα adjusts FAO related genes.

(a and b) Relative *Pparα* mRNA (a) and protein (b) levels in immortalized MEFs treated with ETS (25 μM, overnight). (c and d) PPARα protein levels in 293T (c) and HepG2 (d) cells treated with ETS (25 μM, 24 h). (e) Relative expression of FAO related genes in immortalized

MEFs treated with GW6471 (25 µM, overnight) in the presence of ETS (25 µM, overnight). Statistical analysis was performed using two-way ANOVA with Tukey's multiple comparisons test. (f) Relative *Pparα, Pparβ,* and *Pparγ* mRNA levels in B16F10 cells transfected with siControl or with siRNA against PPARα, PPARβ or PPARγ. (g) Relative expression of FAO related genes in B16F10 cells transfected with nontargeting siRNA (siControl) or two independent siRNAs to PPARα (siPPARα). (h) Relative expression of FAO related genes in B16F10 cells transfected with siControl or with siRNA against PPARβ or PPARγ. For g and h, statistical analysis was performed using one-way ANOVA with Tukey's multiple comparisons test. (i) Relative *Pparα* and *Pparβ* mRNA levels in B16F10 cells treated with ETS (25 µM, 24 h). (j) Quantification of PPARα protein levels for independent biological replicates of the experiment shown in Fig. 3g. Statistical analysis was performed using one-way ANOVA with Tukey's multiple comparisons test. Error bars indicate ±SD (a-i) or ±SEM(j). *$p < 0.05$, **$p < 0.01$, ***$p < 0.001$ and ****$p < 0.0001$.

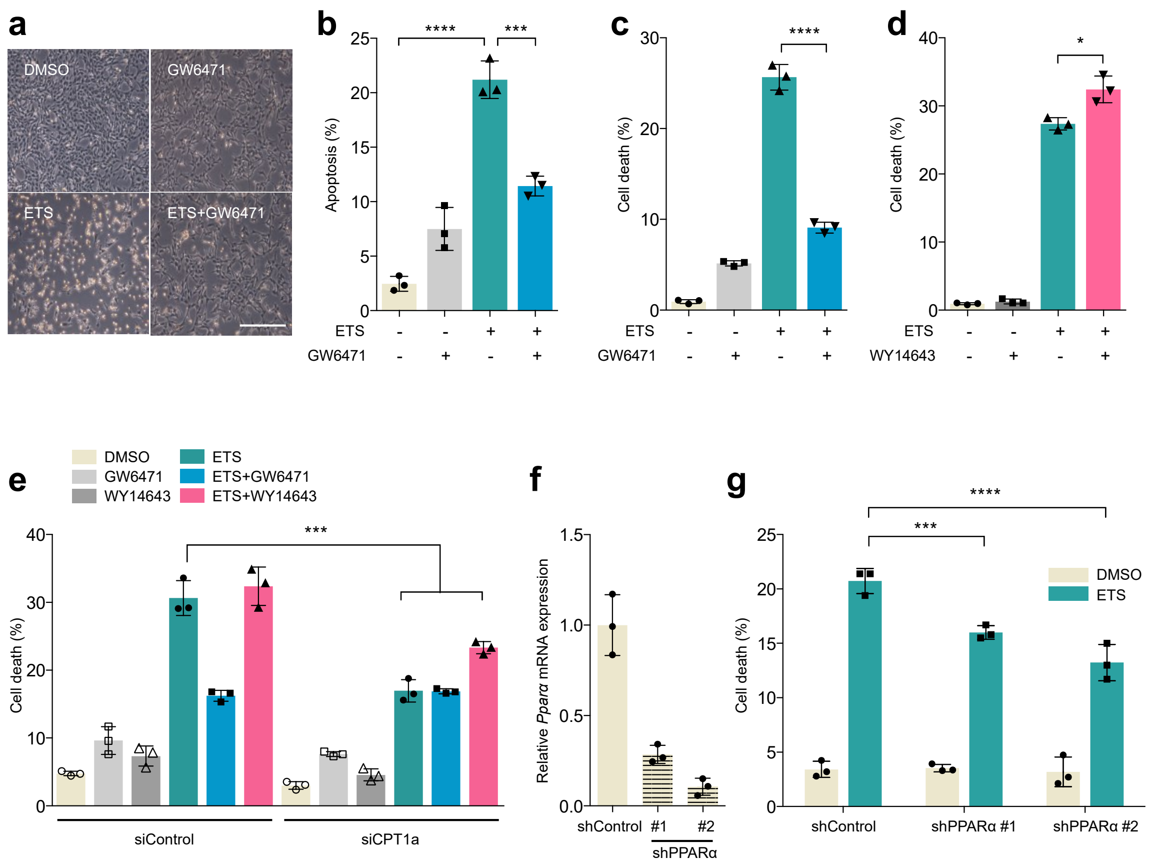

Figure S4. PPARα regulates DNA damage-induced cell death.

(a-c) Microscopy cell images (a) and cell death (b and c) of immortalized MEFs treated with ETS (25 µM, overnight), GW6471 (25 µM, overnight) or both (n = 3). Scale bar represents 500 µm. Cell death was measured by annexin V staining (b) and propidium iodide exclusion assay (c). (d) Cell death of immortalized MEFs treated with ETS (25 µM, overnight), WY14643 (300 µM, overnight), or both (n = 3). Cell death was measured by propidium iodide exclusion assay. (e) Cell death of indicated cells. B16F10 cells transfected with nontargeting siRNA (siControl) or with siRNA against CPT1A (siCPT1A) and than treated with GW6471 (25 µM) or WY14643 (300 µM) and/or ETS (25 µM). (f) Relative *Ppara* mRNA levels in of B16F10 cells expressing a control shRNA or two independent shRNAs to PPARα. (g) Cell death of B16F10 cells expressing a control shRNA or two independent shRNAs to PPARα treated with or without ETS (25 µM, 40 h) (n = 3). Statistical analysis was

performed using one-way ANOVA (b-f) or two-way ANOVA (g) with Tukey's multiple comparisons test (b-f). All error bars ±SD. $*p < 0.05$, $***p < 0.001$ and $****p < 0.0001$.

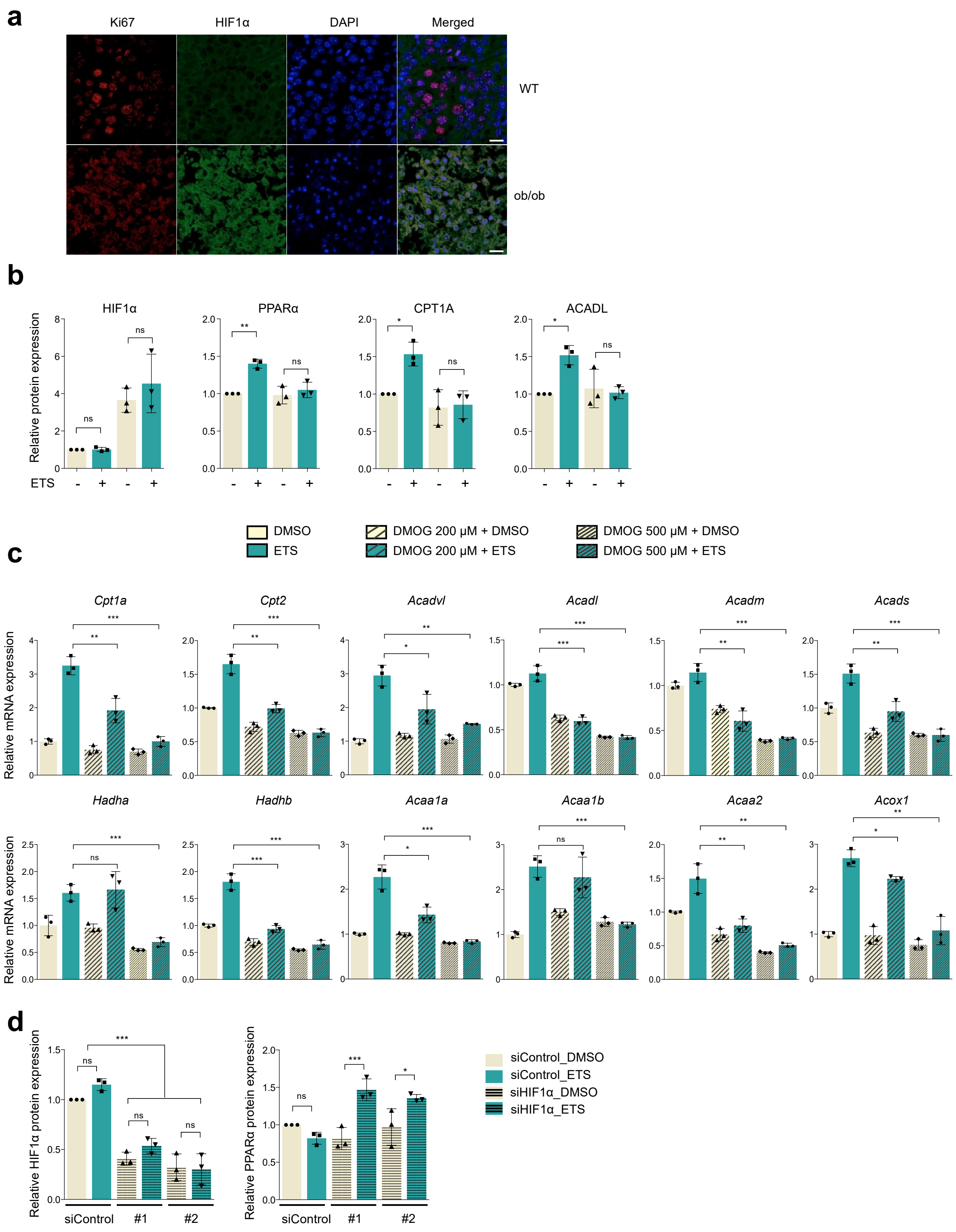

Figure S5. Hypoxia represses PPARα and its target genes.

(a) Immunofluorescence of Ki67 and HIF1α expression in lean and obese B16F10 tumors. Scale bars represent 20 μm. (b) Quantification of HIF1α, PPARα, CPT1A and ACADL

protein levels for independent biological replicates of the experiment shown in Fig. 4d. (c) Relative mRNA levels of FAO related genes in B16F10 cells treated with ETS (25 μM) and DMOG at the indicated doses for 24 h. (d) Quantification of HIF1α and PPARα protein levels for independent biological replicates of the experiment shown in Fig. 4e. Statistical analysis was performed using one-way ANOVA with Tukey's multiple comparisons test. All error bars ±SD. *$p < 0.05$, **$p < 0.01$ and ***$p < 0.001$.

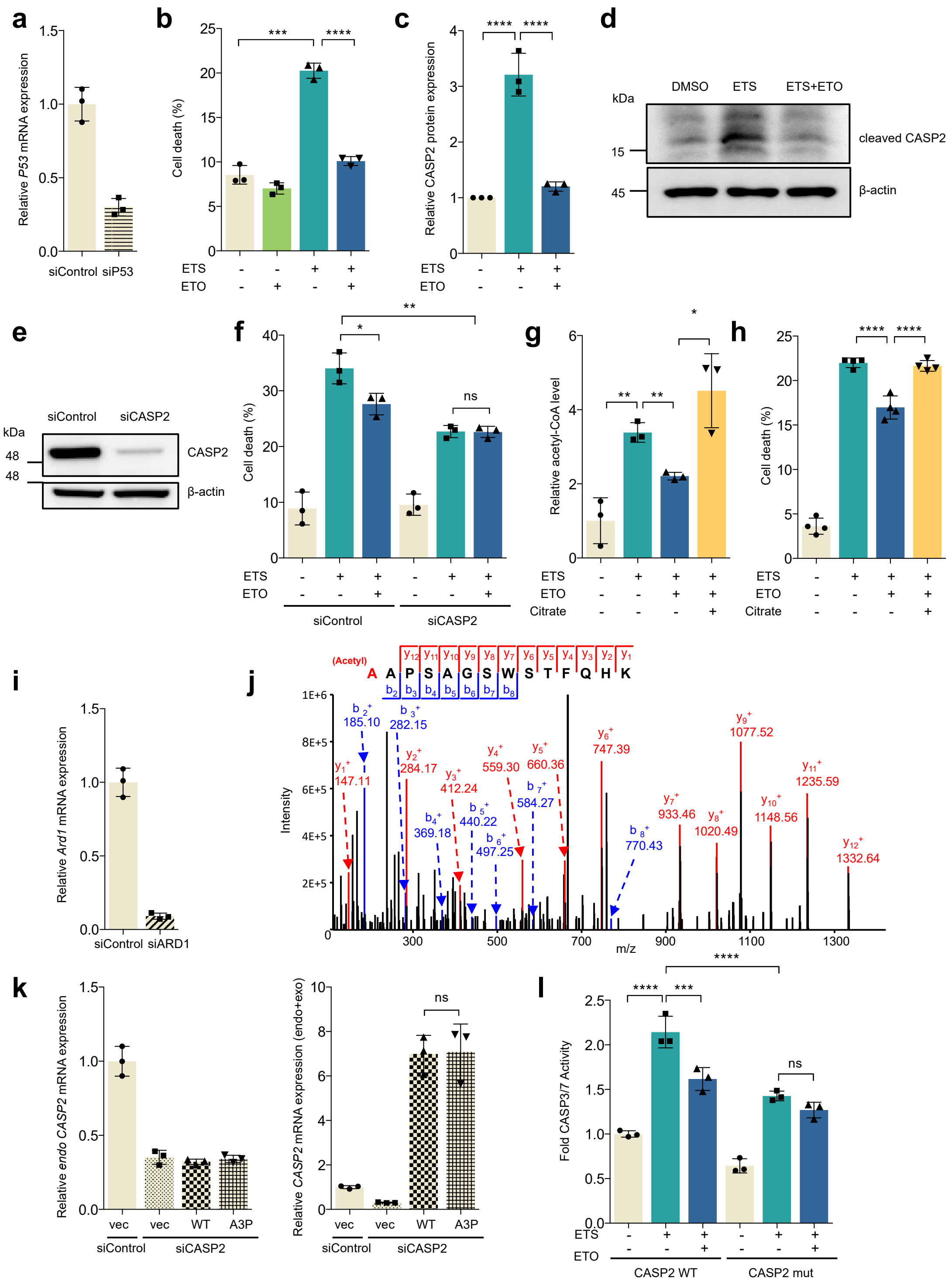

Figure S6. FAO modulates DNA damage-induced cell death by regulating CASP2.

(a) Relative *P53* mRNA levels in cells transfected with siConrtol or with siRNA against P53 (siP53). (b) Cell death of PC3 cells treated with ETS (25 μM, overnight), ETO (200 μM, overnight) or both (n = 3). Cell death was measured by propidium iodide exclusion assay. (c)

Quantification of CASP2 protein levels for independent biological replicates of the experiment shown in Fig. 5b. (d) Cleaved CASP2 expression of B16F10 cells treated with or without ETO (200 μM, 30 h) in the presence of ETS (25 μM, 30 h). β-actin serves as a loading control. (e) CASP2 protein levels in cells expressing a control siRNA or siRNAs to CASP2. (f) Cell death of HeLa cells transfected with nontargeting siRNA (siControl) or with siRNA against CASP2 (siCASP2). Cells were treated with or without ETO (200 μM, 40 h) in the presence of ETS (25 μM, 40 h) ($n = 3$). Cell death was measured by propidium iodide exclusion assay. (g) Relative acetyl-CoA levels were measured in B16F10 cells treated with ETO (50 μM, 24 h) and/or citrate (10 mM, 24 h) in the presence of ETS (25 μM, 24 h). (h) Cell death of B16F10 cells treated with the indicated drugs for 40 h. Cell death was measured by propidium iodide exclusion assay. For f-h, statistical analysis was performed using one-way ANOVA with Tukey's multiple comparisons test. (i) Relative *Ard1* mRNA levels in cells transfected with siConrtol or with siRNA against ARD1 (siARD1). (j) FLAG-tagged CASP2 (C320G) was expressed in 293T cells. Lysates were immunoprecipitated with FLAG magnetic beads. The eluent was performed mass spectrometry. N-terminal peptide corresponding to acetylated caspase-2 is A(Ac)APSAGSWSTFQHK at $m/z = 759.46$. (k) Relative *CASP2* mRNA levels in the indicated cells. HeLa cells were infected with mock vector, hCASP2 WT, or hCASP2 A3P and selected by puromycine (2 μg/ml). Infected HeLa cells were transfected with siControl or with siRNA against CASP2 (siCASP2). Expression of endogeneous CASP2 was determined using CASP2 3' UTR detecting primers (left). Total *CASP2* mRNA expression was analyzed using CASP2 CDS detecting primers (right). (l) CASP3/7 GLO assay of CASP2 WT or CASP2 mutant B16F10 cells. Both cells were transfected with siRNA against CASP2 (siCASP2) and then treated with ETS (25 μM, 24 h), ETO (50 μM, 24 h) or both ($n = 3$). For k and l, statistical analysis was performed using one-way ANOVA with Tukey's multiple comparisons test. All error bars ±SD. *$p < 0.05$, **$p < 0.01$, ***$p < 0.001$ and ****$p < 0.0001$.

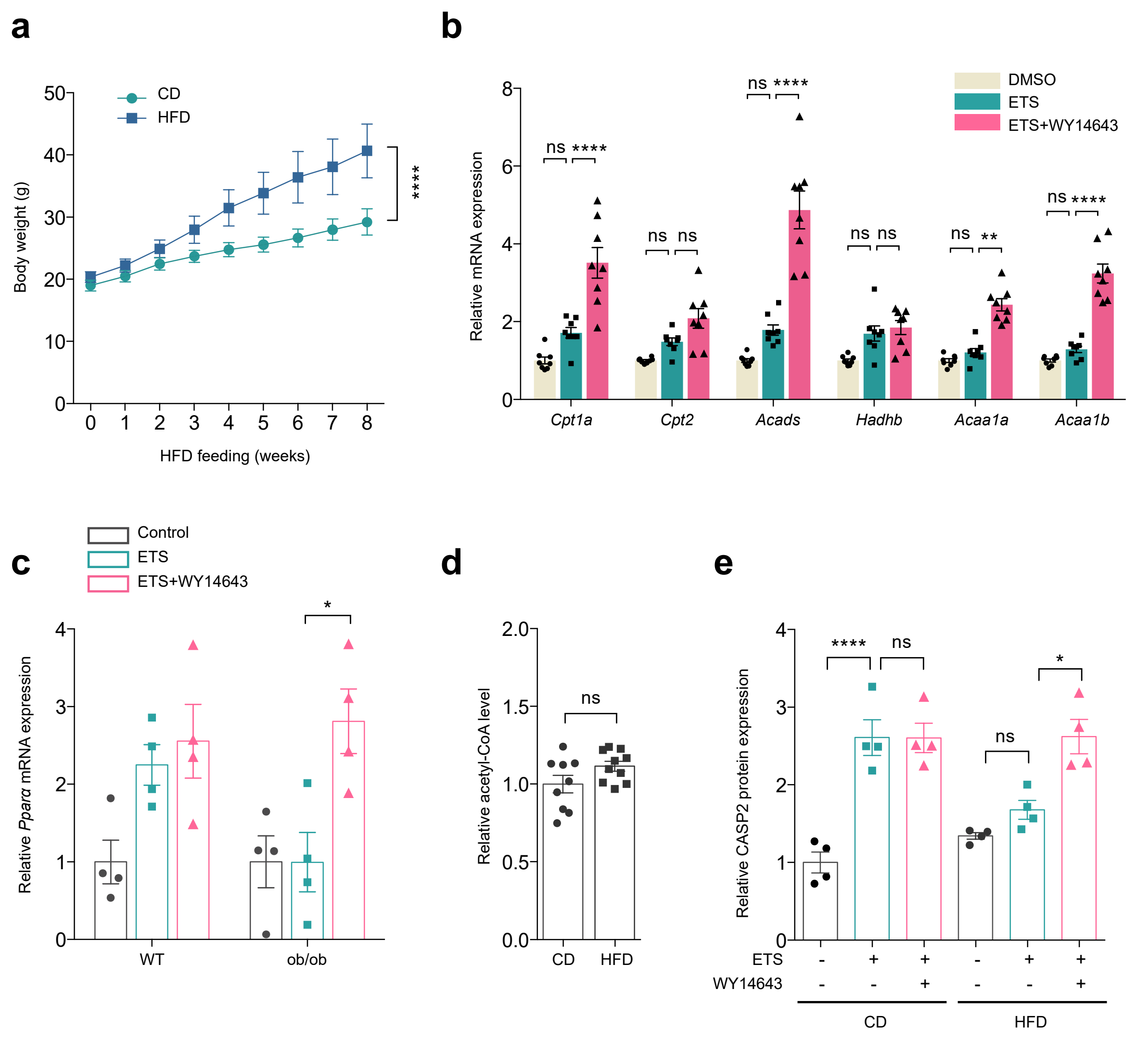

Figure S7. Co-treatment of PPARα agonist with chemotherapeutic drugs induces synergistic effects in obese mice.

(a) Body weights of male mice on CD or HFD feeding. (b) Relative expression of FAO related genes in allograft B16F10 tumors from HFD-fed mice treated with or without WY14643 (40 mg/kg 5 times via intraperitoneal (i.p.) injection) in the presence of ETS (20 mg/kg 5 times via i.p. injection). Statistical analysis was performed using two-way ANOVA with Tukey's multiple comparisons test. (c) Relative *Ppara* mRNA levels in allograft tumors from WT and ob/ob mice treated with or without WY14643 (40 mg/kg 5 times via i.p. injection) in the presence of ETS (20 mg/kg 5 times via i.p. injection). Statistical analysis was performed using two-way ANOVA with Tukey's multiple comparisons test. (d) Relative

acetyl-CoA levels in allograft B16F10 tumors from CD- and HFD-fed mice. Statistical analysis was performed using two-tailed Student's t-test. (e) Quantification of CASP2 protein levels for independent biological replicates of the experiment shown in Fig. 6f. Statistical analysis was performed using one-way ANOVA with Tukey's multiple comparisons test. All error bars ±SEM. $^{*}p < 0.05$, $^{**}p < 0.01$ and $^{****}p < 0.0001$.

Supplementary Table

Primers for qRT-PCR			
**Species**	**Gene**	**Primer forward**	**Primer reverse**
Mouse	*Acsl1*	ATC TGG TGG AAC GAG GCA AG	TCC TTT GGG GTT GCC TGT AG
	Cpt1a	TTG ATC AAG AAG TGC CGG ACG AGT	GTC CAT CAT GGC CAG CAC AAA GTT
	Cpt2	CAA CTC GTA TAC CCA AAC CCA GTC	GTT CCC ATC TTG ATC GAG GAC ATC
	Acadvl	GGC TCT CCA AGG CTG TAT G	ACC ACT GCG ACT TAA CTC TG
	Acadl	TTT CCT CGG AGC ATG ACA TTT T	GCC AGC TTT TTC CCA GAC CT
	Acadm	TCG GTG AAG GAG CAG GTT TCA AGA	AAA CTC CTT GGT GCT CCA CTA GCA
	Acads	GAG CTT GGC TGC CTC TTT AC	CAT GGG AAC AGC ACT GAG AG
	Ehhadh	CAG ATG AAG CAC TCA AGC TTG	ACC TTG GCA ATG GCT TCT GCA
	Echs1	GAA CAC ATC GTC TCT CCG CC	TGA AAG TTA GCA CCC GAG GC
	Hadha	TGC ATT TGC CGC AGC TTT AC	GTT GGC CCA GAT TTC GTT CA
	Hadhb	GAT AAG ACT CCA GCT CAC ACT G	CCA GAA GCT ATC AGA CCA ACA G
	Acaa1a	GAG AGT GAG AAA GCC AGA GAC	CTG CCG TGA AAT GCC AAA C
	Acaa1b	GAG ACT GCC TGA TTC CTA TGG	GCA CAA TCT CAG CAT GGA AG
	Acaa2	TGT TCT AAA GAT GCT GAG GTC G	TTG ATC CGT TAA TCC TGC CC
	Acox1	TGC CTT TGT TGT CCC TAT CCG TGA	TTA CAT ACG TGC CGT CAG GCT TCA
	Pparα	CAT TTC CCT GTT TGT GGC TG	ATC TGG ATG GTT GCT CTG C
	Ard1	GCA TCT CTA TTC CAA CAC CCT C	CAT ACG CAT CTT CCC CAT CTG
	Lipg	TTC GCA ACT ACC TGT CTC AAC CCA	TCC AGC CAT CCT GAC ACT TGT GAA
	Acot2	CCC CAA GAG CAT AGA AAC CAT	CAT AGC AAG GCC AAG TTC AC
	Acot3	TGG AAT TGG AAG TGG CCT TCT GGA	AAC CTG TTA CCT GAG GGT GAC TGA
	Scd3	CCG AGA AGC TGG TGA TGT TC	AGC GTA TCT CAG TAA AGT GGC
	Abca2	TCT ACT CAC GCC TCA AAA GC	TTC ATG CCT CCA GAC AAC G
	β-actin	AGC CAT GTA CGT AGC CAT CC	CTC TCA GCT GTG GTG GTG AA
Human	*CASP2 (CDS)*	TTCTATGTGACCAGACTGC	AGTTTCCCATCCACACCATAG
	CASP2 (3'UTR)	TCATCTCTGCCTTTGAGTGTG	ACTTCACTTGTCAGGCTCTTC
	β-actin	CTACGTCGCCCTGGACTTCGAGC	GATGGAGCCGCCGATCCACACGG
